# Spatial-Temporal Genome Regulation in Stress-Response and Cell-Fate Change

**DOI:** 10.3390/ijms24032658

**Published:** 2023-01-31

**Authors:** Jekaterina Erenpreisa, Alessandro Giuliani, Kenichi Yoshikawa, Martin Falk, Georg Hildenbrand, Kristine Salmina, Talivaldis Freivalds, Ninel Vainshelbaum, Jonas Weidner, Aaron Sievers, Götz Pilarczyk, Michael Hausmann

**Affiliations:** 1 Latvian Biomedicine Research and Study Centre, LV1067 Riga, Latvia; 2 Istituto Superiore di Sanita Environment and Health Department, 00161 Roma, Italy; 3 Faculty of Life and Medical Sciences, Doshisha University, Kyoto 610-0394, Japan; 4 Institute of Biophysics, The Czech Academy of Sciences, 612 65 Brno, Czech Republic; 5 Kirchhoff Institute for Physics, Heidelberg University, 69120 Heidelberg, Germany; 6 Faculty of Engineering, University of Applied Science Aschaffenburg, 63743 Aschaffenburg, Germany; 7 Institute of Cardiology and Regenerative Medicine, University of Latvia, LV1004 Riga, Latvia; 8 Doctoral Study Program, University of Latvia, LV1004 Riga, Latvia; 9 Institute for Human Genetics, University Hospital Heidelberg, 69117 Heidelberg, Germany

**Keywords:** dynamic genome organization, epigenetic interactions, heterochromatin and self-organization, organizational and functional networks, gene activity oscillations, transposon-effected regulation, fluorescence microscopy, topological genome analysis, database pattern analysis, nucleotide k-mers

## Abstract

Complex functioning of the genome in the cell nucleus is controlled at different levels: (a) the DNA base sequence containing all relevant inherited information; (b) epigenetic pathways consisting of protein interactions and feedback loops; (c) the genome architecture and organization activating or suppressing genetic interactions between different parts of the genome. Most research so far has shed light on the puzzle pieces at these levels. This article, however, attempts an integrative approach to genome expression regulation incorporating these different layers. Under environmental stress or during cell development, differentiation towards specialized cell types, or to dysfunctional tumor, the cell nucleus seems to react as a whole through coordinated changes at all levels of control. This implies the need for a framework in which biological, chemical, and physical manifestations can serve as a basis for a coherent theory of gene self-organization. An international symposium held at the Biomedical Research and Study Center in Riga, Latvia, on 25 July 2022 addressed novel aspects of the abovementioned topic. The present article reviews the most recent results and conclusions of the state-of-the-art research in this multidisciplinary field of science, which were delivered and discussed by scholars at the Riga symposium.

## 1. Introduction

Except for polyploid cells in some organs, all cells of the multicellular higher organisms have the same amount of DNA independently of their physiological role and dependent on the biological species. In humans, about 2 m of DNA contains the linearly sequenced genetic information, while there are about 250 types of tissue and approximately 400 cell types [1] with different phenotypes and functions. The genetic linear information embedded in the DNA sequence should contain all information that is necessary for the specialized functioning of all these cell types; thereby, mechanisms of switching the genes on and off, and other genome functions must be also included as well as motifs inducing a preferred folding and thus accessibility or inaccessibility of chromatin. The protein apparatus required for the given functioning contains components that are generally needed for functioning of all cell types, and others that are only necessary for the cellular specialization. This means that cells must be flexibly able for appropriate on- and off-switching. In this context, the main questions are where the control towards this normally monodirectional diversification comes from, how the adaptation of cells to the environment appropriately functions, either on a daily basis (e.g., short exposures of chemicals, radiation, etc.) or in the case of lethal challenge, and how cells inherently change their fate, e.g., from normal differentiation to tumor genesis and vice versa. Since a cell nucleus is a complex system that can only react as a whole [2], all these questions are linked together with the same enigma related to the genesis of the organism and also to cancer with its extreme resistance to lethal therapies, e.g., ionizing radiation treatment.

The basic question is “How can the specificity of biological interactions arise without the participation of ‘intelligent agents?’” The general answer is “By self-organization.” This concept was first developed in chemistry and physics and then applied to various morphogenetic problems in biology over the past century, and it is now beginning to be applied to the organization of the living cell [3] and attempted to be adjusted to the genome organization [4,5]. Some features of genome self-organization, such as chromatin dynamics, phase separation (hetero- and euchromatin), architectural elements (nuclear lamina), and polymer–polymer interactions [5], still have a mainly phenomenological character but allow for a physically motivated view of biological regulation driven by spatiotemporal heterogeneity modulating the intrinsic stochasticity of the system at hand.

In this article, we show that physical mechanisms are penetrating all these regulations and help to depict a unifying, albeit tempting, explanation of genome expression regulation. This work stems from the contributions of different research groups, and thus it is important to stress some basic hints to find the common track latent to the apparent patchiness of the different chapters. All the different contributions try to answer the same basic question formulated above.

This question has arisen many times along with the development of biological sciences. Probably the first modern satisfactory (albeit confined to the microscopic molecular level) answer was the Monod–Wyman–Changeux model of the allosteric effect [6]. In the case of allosteric signaling, the specificity is a consequence of the strict spatial constraints imposed on protein molecule configuration by the extreme distance-dependent constraints of noncovalent bonds. The “action at distance” exerted on the active site by the binding of specific effector molecules at a distal allosteric site acquires its specificity by the need of satisfying a set of very strict configuration constraints. This very successful model is the physical foundation of all the subsequent developments of molecular biology allowing us to take for granted that a specific effector molecule can recognize its target, thus enabling the establishment of intermingled molecular pathways assuring the specificity of biological regulation at large. This kind of reductionist thinking where the only causative relevant level is the molecular one enters into crisis when considering the multiscale character of biological systems. As highlighted in Section 2.1, the compaction of human DNA molecules of enormous linear size (approximately 2 m in a diploid cell) into the micron scale of the cell nucleus, make it unfeasible to rely on such regulation. This asks for something different: we must look somewhere else for giving a physically plausible explanation to biological regulation. Individual chapters of this work correspond to different viewpoints on the genesis of biological regulation specificity. A common starting point is the inescapable link, set forth by nonlinear thermodynamics, between symmetry breaking and information (and thus specificity) generation [3,7]. Specificity can only arise in a nonuniform (and thus asymmetric) landscape, that is to say, that biological matter (e.g., genome spatial structure at different scales) is a sort of an aperiodic crystal (see Section 2.7) with the possibility to individuate reference points generating positional information (see Section 2.2). The signatures of such positional information supporting regulation specificity can be found on different scales from molecular (see Section 2.5) to supramolecular architectures (Section 2.3 and Section 2.6), as well as in the time dimension, up to critical self-organization found in cancer (see Section 2.2 and Section 2.4).

The existence of a rich multiscale spatial structure, dynamic in time, makes it possible to adapt to external forces with a consequent rewiring/reshaping of biological systems (see Section 2.8 and Section 2.9), thereby closing the circle of biological regulation, which by definition must be able to “map” the microenvironment.

## 2. Sessions and Presentations

In this section, divided by the contributions the conference was addressing, state-of-the-art and novel results, their interpretation, as well as the experimental conclusions will be presented.

### 2.1. The Guardians of Stability Are the Same That Initiate Revolutions: The Oscillatory Principle of Gene Expression Dynamics (Presented by A. Giuliani)

Maxwell’s demon is an imaginary entity proposed in a thought experiment created by James Clerk Maxwell in 1867 in which he suggested how the second law of thermodynamics might hypothetically be violated. In the thought experiment, a demon controls a small door between two chambers of gas. As individual gas molecules approach the door, the demon quickly opens and shuts the door so that only fast molecules can pass into one of the chambers, while slow molecules cannot. Because faster molecules are hotter, the demon’s behavior causes one chamber to warm up and the other to cool down, thereby decreasing entropy and violating the second law of thermodynamics.

This mechanism implies the presence of an “intelligent agent” (demon) that alters the natural fate of the system toward an inescapable entropy increase. Many scholars analyzed the different physical implications of such a model (e.g., trying to evaluate the energy spent by the demon to carry out its work and consequently saving the second law of thermodynamics). The issue is much more serious than a scientific joke: almost the totality of biological explanations follows a Maxwell demon style of reasoning with specific effectors (e.g., proteins, transcription factors) that act as intelligent agents thanks to the specificity of their mechanism of action. In some other cases, the need of a Maxwell demon hypothesis is considered the signature of a “preliminary” status of an explanation that asks for being substituted by a more refined (and physically plausible) theory [8]. This last attitude has been the one adopted in this chapter for the case of genome expression regulation, trying to sketch a phenomenological, albeit physically reasonable, model capable of both maintaining homeostasis and the onset of massive reorganization of genome expression profile. This approach allows going further than the local Maxwell demon explanations still prevailing in biological literature [9].

In the following, we show a very peculiar feature of biological systems, namely, that the mechanisms involved in maintaining homeostasis and those responsible for massive changes are one and the same [10]. This peculiarity stems from the concept of self-organized complexity (SOC), a fundamental organizing principle deriving from statistical mechanics and subsequently applied in a wide range of scientific fields [11].

#### 2.1.1. Setting the Scenes

(a) Biological scene (size matters):

There are two main fundamental physical difficulties in achieving large-scale coordinated control on a gene-by-gene basis in both the cases of “business as usual” (i.e., homeostasis, where any cell type must keep a largely invariant gene expression profile) and cell fate determination (i.e., differentiation, when the entire genome is traversed by an expression change encompassing thousands of gene products). The first difficulty is related to the lack of a sufficient number of regulatory molecules in a cell to reach a stable thermodynamic state. The low copy number of specific regulatory molecular species provokes stochastic noise [9,10], thereby inducing a substantial instability of gene product concentrations. The second one derives from the huge linear dimension of the human DNA molecule (around 2 m) with respect to the cell nucleus that makes chromatin very far from a Turing-like string freely accessible by regulatory molecules at the single-gene level. This extreme compression generates a stunning complex organized structure of chromatin at different orders of magnitude that makes the problem of finding a specific gene sequence for a regulatory Maxwell demon agent (e.g., a transcription factor) analogue to finding a needle in a haystack. From a purely theoretical point of view, these massive phenomena ask for considering genome expression regulation an evolution toward a preferred (minimal energy) attractor corresponding to a specific configuration of the system in the space spanned by the relative abundance of a huge number of molecular players [9,12]. This can happen in the presence of a general “energy field” that shapes a rugged landscape where the valleys correspond to attractor states, according to the visionary intuition of Conrad Waddington’s “epigenetic landscape” [13]. This picture implies a discrete (and relatively low) number of possible cell fates. This is exactly the case: a huge number of genetic elements (in the order of 10^4^ considering even nonstructural genes), each potentially varying in expression level within three orders of magnitude, give rise to ~400 cell types and subsequently ~250 different tissue types in humans [1], with each cell type corresponding to an attractor set. That is to say that an “attractor-like” dynamics is made necessary by the simple observation of organism composition.

**Figure 1:**
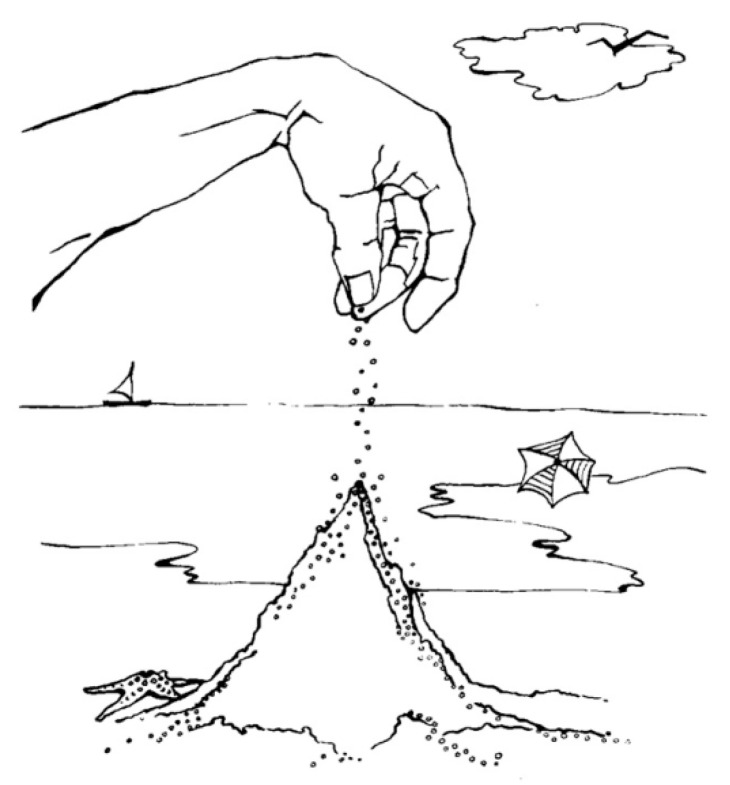
A pictorial representation of the SOC (sandpile model): the grains are gently added grain by grain to the sandpile (continuous small perturbations from the microenvironment), provoking small avalanches that keep invariant the sandpile slope (homeostasis). Occasionally, by a sort of domino effect, when avalanches cooperate, the core of the sandpile is affected and the sandpile goes down (cell-fate transition).

(b) Physical scene (sandpile tales):

The model of “self-organized complexity” (or “self-organized criticality,” when the focus is on the peculiar situation of having an attractor state located in a critical position on the edge of chaos) or SOC (both interpretations give rise to the same acronym) was developed by Per Bak and colleagues [14]. The idea of SOC stems from the so-called sandpile model shown in a pictorial way in Figure 1.

Think of pouring sand very slowly onto a flat, circular surface. At first, the grains stay close to where they land and very soon start to accumulate; creating a pile that has a gentle slope. Going on with the experiment, somewhere on the pile, the grains slide down, causing a small avalanche. As we add more sand, the slope of the pile steepens, and the average size of avalanches increases. The pile stops growing when the amount of sand added balances the amount of sand falling off the edge of the circular surface. At this point, the system reaches the critical state that is a stable attractor: the continuous avalanches are counterbalanced by the added sand, while the height and shape of the pile remains the same. Occasionally, an added grain can cause a large catastrophic avalanche by a chain reaction involving progressive avalanches falling down to the base and thus flattening the entire sandpile (long-range correlation, typical of transitional states). The chain reaction can be imagined as a branching process invading a large part of the pile: during the slide, the grain hits other grains, causing progressively larger avalanches [14]. The conundrum is solved: the equilibrium state is, at the same time, an attractor, i.e., a stable state, and a critical state prone to catastrophic changes. The frequent and small avalanches happen at the periphery (liquid-like phase) of the system, while catastrophic (very rare) events involve the core of the sandpile. This distinction into a highly variable (fluid) periphery taking care of both dissipation of external perturbation preserving the largely invariant (crystalline) core and favoring transitions is very common. The same fluid-phase in charge of protecting the core is the place where massive changes start.

#### 2.1.2. Gene Expression Regulation at the Genomic Scale (Putting SOC at Work)

**Figure 2:**
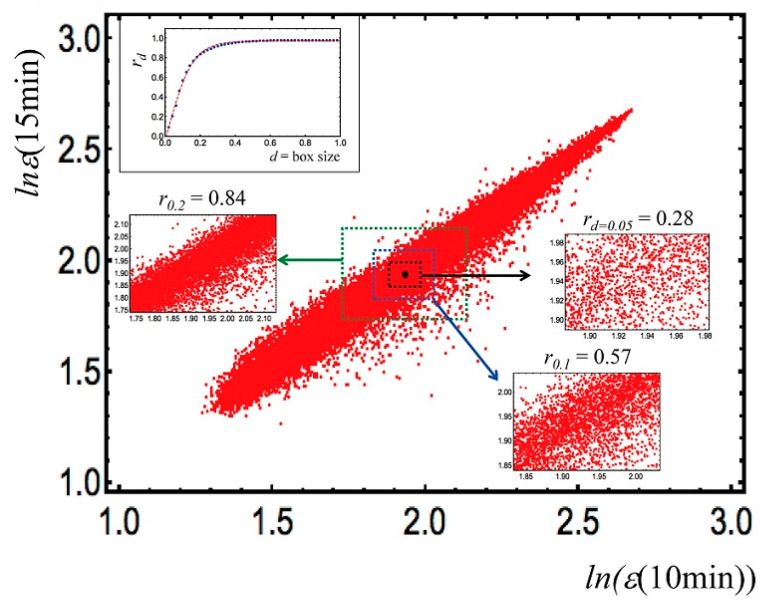
The axes of the figure correspond to the gene expression profile of two independent samples of the same cell kind (MCF 7 cancer cells). The vector points are the single gene expression levels expressed in logarithm units. The expression profiles are very strongly correlated across the whole genome (Pearson r = 0.98). Snapshots at smaller scale (d = range of variation) show progressive decrease in correlation with increasing detail (lower d). Top left inset shows the reaching of a plateau correlation at d = 0.5.

The continuous small avalanches keep the sandpile slope invariant and dissipate (without consequences for the global stability of the sandpile) the continuous perturbations coming from the environment (addition of sand grains). Stability, in a continuously varying environment, needs a relentless oscillation of the system; this “dynamical stability” is maintained by periphery elements that prevent the perturbation to affect the core of the system. In the case of genome expression, these elements are genes endowed with an elevated spontaneous variability (periphery of the attractor) orthogonal to the main gene expression variance profile (cell-type attractor, core of the sandpile, PC1, first principal component of expression variance). Figure 2 gives a graphical representation of this condition.

It is evident from Figure 2 that the invariance of the entire genome expression profile (Pearson r = 0.98) at the global scale relies upon oscillations (departures from the main diagonal) at smaller scales (small avalanches ensuring global scale homeostasis).

**Figure 3:**
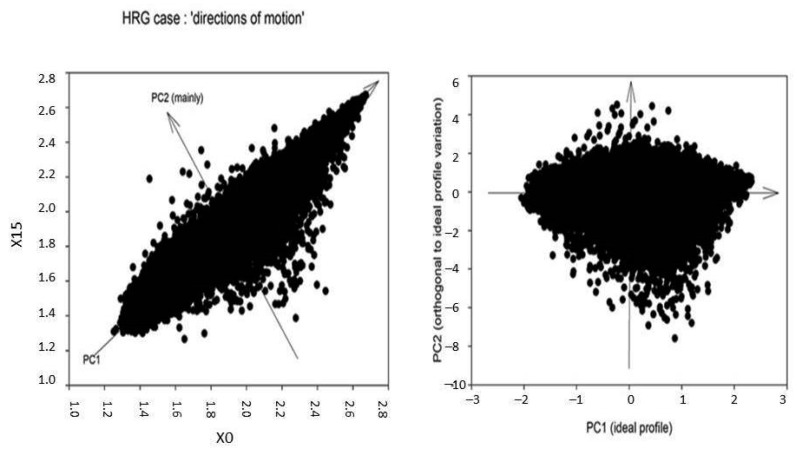
Left panel, the genome expression profile at X0 (equilibrium position) and X15 (transition): a direction of motion orthogonal to the main component (PC2) drastically increases in size, invading the core of the system. This invasion provokes the increase in variability of genes that in the equilibrium state were practically coincident with the line of identity (cell-type attractor). Right panel, the same situation in the principal component space expressed in z-scores.

When in presence of a transition, provoked by heregulin (HRG) [10] the same genes endowed with the role of keeping homeostasis increase their oscillation (bigger avalanches) and the direction of motion orthogonal to the main attractor (PC2) experiences one order of magnitude increase in power, provoking a phenotypic transition (Figure 3).

It is worth noting that this condition of critical equilibrium, which allows for both homeostasis and transitions by the same basic mechanism, is shared by many biological systems; proteins are the most studied ones [13].

Over the last two decades, the widely accepted paradigm of a well-defined three-dimensional structure as a necessary requisite for proteins to properly function was abandoned. The discovery that almost all the eukaryotic proteins have intrinsically disordered patches and that this disorder was necessary for protein physiology drastically changed the canonical paradigm. Proteins live in a microenvironment continuously perturbing their native structure, and the entities of these perturbations are of the same order of magnitude as intermolecular forces responsible for their 3D configuration. This makes it necessary to dissipate this “extra energy” to keep the structural core invariant, and this is exactly the role of more flexible (disordered, liquid-like phase) patches [15]. At the same time, proteins must respond to “physiologically relevant” stimuli from their microenvironment by changing their configuration in accordance to these stimuli. A classic example is the allosteric phenomenon: a relevant signal (e.g., the partial pressure of oxygen in the case of hemoglobin) is sensed by a part of the structure (allosteric site) and the information (in the form of a configuration transition) must be transported to the active site distant from the allosteric site. This happens by the action of the so-called entropic reservoir [15] made of the flexible parts of the molecule that both dissipate thermal noise and drive allosteric transition when needed, thanks to the “invasion” of the protein structural core by a wave of coordinated motion.

The stability of complex systems made of a set of intermingled relations among their elements, like gene expression or protein molecules, and embedded in a continuously fluctuating environment derives from principles very different from simple “resistance to change”. These systems must not only maintain their identity against the disrupting effect of noise but also must actively rearrange when in the presence of “information-relevant” stimuli coming from their environment. The discrimination of irrelevant (to be dissipated) and relevant (that necessitates a configuration change) is mediated by the juxtaposition of a fluid (highly variant) and a crystalline (largely invariant) phase. The liquid-like elements act as both “guardians of stability” dissipating the excess of energy coming from noise and as “revolutionary agents” letting the relevant stimuli to re-wire the system.

### 2.2. Genome Regulation by Positional Information in Space and Time (Presented by J. Erenpreisa)

The presentation of Jekaterina Erenpreisa (J.E.) was based on the current knowledge of the thermodynamics of open unstable systems, whose principles were developed by physicists in collaboration with biologists in the past century (Stuart Kauffman, Ilja Prigogine, Conrad Waddington and others). These studies contributed to a fertile interaction between statistical mechanics and systems biology (prominent names related to these developments are: Sui Huang, Alessandro Giuliani, Denis Noble and others). The talk of J.E. involved her own experimental results in studying embryos, cell differentiation and cancer during the last 40 years, which much corresponds to the rules of predetermined chaotic regulations in the living systems [16]. The approach started from the idea of a genome field organized by heterochromatin [17], which goes back to the theory of biological field developed by A. Gurwitz, W. Nagl and F.A. Popp (“electromagnetic field”), A. Lima-de-Faria (“chromosome field”), L. Wolpert (“morphogenic field”) and of heterochromatin studies by E. Heitz, S. Ohno, I. Zhimulev, Y. Hiraoka, L. Manuelidis, D. Comings, and others, in the past century. In the 1980s and 1990s, J.E. investigated this problem using DNA cytometry-based quantitative image analysis of cell nuclei [18,19,20,21], mostly in collaboration with Alexander Zhukotsky. These studies shed light on the nature of the genome networks, revealing heterochromatin-organized cell nucleus networks and the radial gradient in its dynamic organization.

In the last decade, the genome studies by J.E. were further promoted in collaboration with Alessandro Giuliani [7], Michael Hausmann [22] and with the group of Kenichi Yoshikawa, who formulated the idea of genome field realization through the self-organization of gene expression [23].

In the current communication, J.E. is attempting to update the knowledge and her group data on the relationship between spatial and temporal regulation of the genome, based on its oscillatory character stemming from the theory of dissipative systems [24,25].

**Figure 4:**
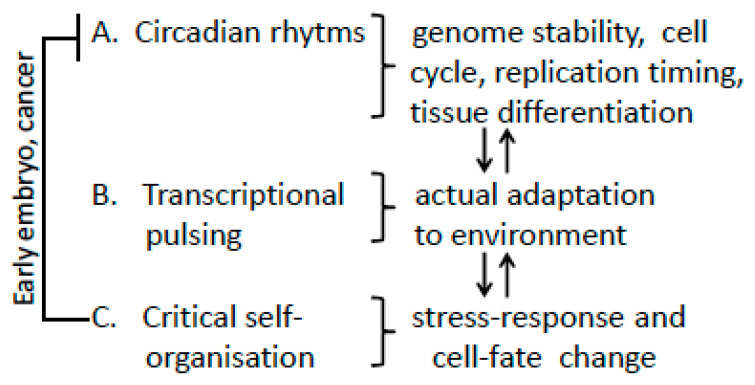
A scheme of three time–space-related regulation modes of cell functions. (**A**) By circadian 24 h clock—a molecular oscillator sub-driving the regulation of the cell cycle (pace and checkpoints) and replication timing, which is setting the epigenetic (tissue-specific) chromatin compartments. In general, this oscillator cares for the genome stability and its robust functions. (**B**) Transcription pulsing (bursting), whose frequency is determined by tissue-specificity but volume of individual gene transcripts, is adapting by feedback to environment demands. A special type of transcriptional pulsing—the immediate stress response—is a prerequisite of transition from the small fluctuations supporting functional homeostasis to (**C**) critical self-organization of the whole genome, which is occurring very rapidly, using a bistable phase transition and changing cell fate. This type of regulation is observed in an early embryo, embryonic stem cells, and in cancer (related to polyploidy) and acts along with deterioration of circadian clock.

After years of dispute, the nucleome molecular scientists finally reached a consensus that heterochromatin (HR) contacts play a primary role in the large-scale genome [26,27] organization, thus confirming the data obtained by a pleiad of the abovementioned scientists. Here, J.E. analyzed the three types of the spatial-temporal genome regulation (Figure 4). Classified as A, B, and C, they arbitrary reflect the relationship between the processes driven by linear and unstable thermodynamic: from “order” (A), through “bistable switch” (B) to “at the end of chaos” (C). The thermodynamic parentage of cancer and early embryo (in support of the embryonal theory of cancer) originated in the 19th century [7,28] is outlined.

As well established in the past, during the interphase, chromosomes are largely positioned in a radial order between the nucleolus and nuclear envelope [29]. The hypothesis postulated by J.E. in 2014 was: The tissue-specific information is created by the latitude positions of silencing constitutive heterochromatin cluster network set by replication timing upon the radial chromosome longitude as the “globe-map address”.

The stability of tissue differentiation, determined by replication timing and executed in a regular cell cycle when it is being established, is supported by a circadian molecular clock (CC). The CC, in turn, is dependent on the constant 24 h rhythm of the Earth rotation around its axis. As the Earth was created 4.5 billion years ago, it is not surprising that CC was already encoded in cyanobacterial cells at the atomic level expressed in a 10 nm diameter biomolecule [30]. In multicellular organisms, the circadian pacemaker is a molecular oscillator composed of two groups of a few proteins that regulate cell cycle checkpoints and coordinate DNA replication with differentiation pace in normal development starting from embryo invasion into its mother’s endometrium [31]. However, this stability is not supported in the preimplantation embryo, embryonic stem cells, and likely cancer, which are regulated by self-organization to a larger extent (Figure 4) [7,32]. This seeming paradox may be explained that in this way, at the “edge of chaos,” the adaptability to unforeseen challenges increases the chance of survival of the most precious biological material—the embryo. For the employment of the same adaptation instrument in cancer biology, see Section 2.4.

Transcription pulsing is another kind of rhythm, with an irregular ultradian period (~20–40–120 min). Its collective frequency determines the cell type and depends on enhancers, while the transcription amplitude of individual genes depends on the promoters [33,34]. The clusters of enhancers linked to gene promoters create transcription hubs that represent an inner compartment (alveoli) of the tissue-specific heterochromatin network. The two types of contact between chromosomes form the functional nuclear network: on one hand, sticking (kissing) heterochromatin knots create suppressive positional information; on the other hand, the repulsing forces of the transcribing active DNA loops joined with transcription hubs foster the activating network (as alveoli of the former). These two mechanisms specify positional information [21,35,35].

In the cell nucleus, transcription pulsing, starting from the nucleolus, is made possible by the radial-concentric organization of the structural participants cooperating with the elastic cytoskeleton. For more details of this dynamic organization, see Section 2.3 and [36].

**Figure 5:**
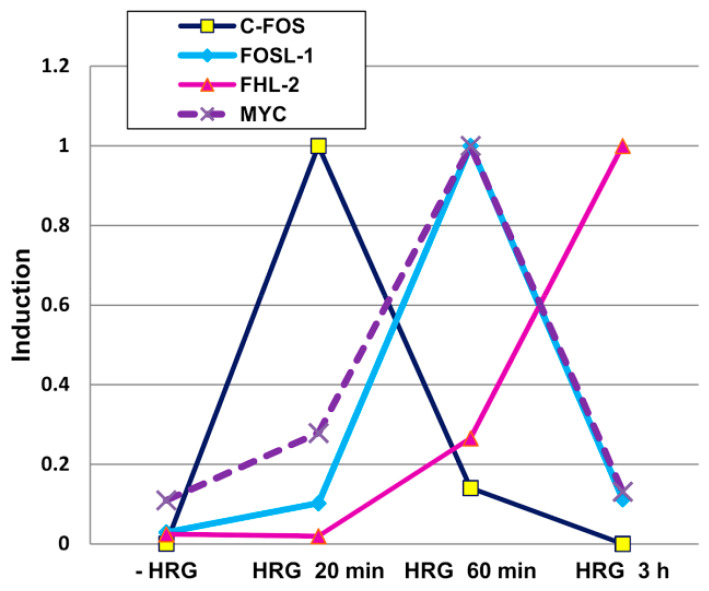
qPCR evaluation of the transcription in the dynamics of HRG treatment. Biphasic expression of the early response genes tested at four time points and presented in relation to the maximum averaged expression (1 unit on *Y*-axis) of each of the four tested genes. Representative graphs of two independent experiments. (Note: This figure and a slightly modified figure legend were originally published under CC-BY license in [41]).

Particularly large transcription bursts synchronizing cell populations are characteristic of and can be induced in the early stress response. The participating genes are bivalent (contain both activating H3K4me3 and repressing H3K27me3 histone modifications at their promoters) and code for transcriptional factors, such as FOS and its ligand, c-myc, opening the chromatin, and multiple secondary targets. The strongly synchronized transcription oscillation of stress-response genes precedes cell-fate change, including desperate attempts at survival in some systems of lethal challenge. In such populations (ascitic of rat hepatoma Zajdela or mouse Sa180 cells firmly plugged in a vial), a few large amplitudes but fading waves of synchronized transcription were observed preceding apoptotic cell death. They were accompanied by the increased DNA superhelicity alternating with the emergence of the initially reparable single-strand breaks in the antiphase [37,38]. It cannot be excluded that these fluctuations at the brink of death are part of the mechanism of the early apoptosis reverse, termed “anastasis” [39]. In another model of cell-fate change in breast cancer MCF7 cells towards redifferentiation by heregulin (HRG) [40,41], the high biphasic activation of stress-response genes was registered (Figure 5).

**Figure 6:**
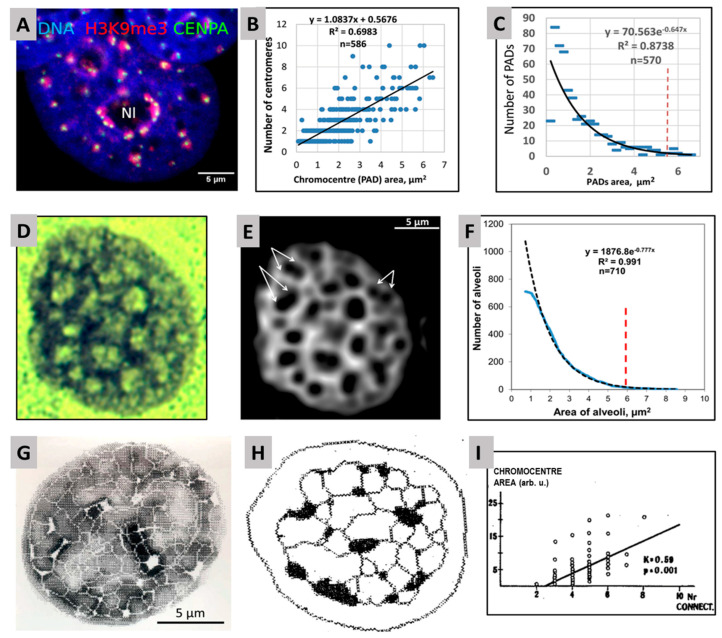
Image analysis data showing the causal relationship between PAD chromocenter clusters and their network in interphase of untreated cell nuclei. (**A**,**B**) Confocal image of PAD clusters stained for repressive landmark H3K9me3 and centromere protein (CENPA) showing colocalization and also linear relationship between the PAD area and the number of centromeres; (**C**) negative exponential relationship between PAD numbers and their area in MCF-cells; (**D**–**F**) similarly negative exponential relationship between the number and size of the alveoli of the heterochromatin network (revealed by preferential HR-staining method and image filtration) in the same MCF7 cells (double alveoli are arrowed); (**G**–**I**) cell nucleus network of chicken embryo femur cartilage stained by toluidine blue stoichiometrically for DNA determination; (**G**) skeletonized chromatin network and chromocenters are overlaid onto the half-tone image; (**H**) another nucleus image showing the overlay of separately discriminated dense chromocenters and skeletonized chromatin network; (**I**) positive relationship between the area of the chromocenters and the number of branches from them confirming the origin of the network by the chromocenter clustering. (Note: This figure and a slightly modified figure legend was originally published under CC-BY license in [36]).

In turn, a cell-fate change requires emergent self-organization by critical global phase transition. In the mentioned model of breast cancer MCF7 cells induced by HRG, an acceleration of transcription involving thousands of genes was found in a narrow time period—15 to 20 min after HRG action [9]. Further, we inquired if HR is involved in this critical phase transition. An important work that J.E.’s laboratory has recently published [41] revealed the scale-free distribution of the clusters containing pericentromere-associated constitutive heterochromatin domains (PADs) in cell nuclei (Figure 6A–C). For details, see Section 2.3. This scale-free distribution of PAD clusters indicates their ability and necessity to create dynamic networks [14]. Indeed, the heterochromatin-PAD-clustered networks were shown by microscopy as possessing similar parameters in various types of chicken, rat, and human cells with five chromatin imaging methods (including superresolution microscopy) [36], with examples in Figure 6D–I.

The splitting of PAD clusters between 15 and 20 min after HRG action in MCF cells described in detail in Section 2.3 occurring simultaneously with the emergent transcription avalanche under the same conditions and terms, confirmed the role of HR-determined positional information presented as a network in regulation of cell-fate change by critical self-organization.

### 2.3. Scale-Free Organization of Pericentric Chromatin Domains in MCF7 Breast Cancer Chromatin Nuclei and the Impact of HRG Treatment (Contributed by T. Freivalds)

**Figure 7:**
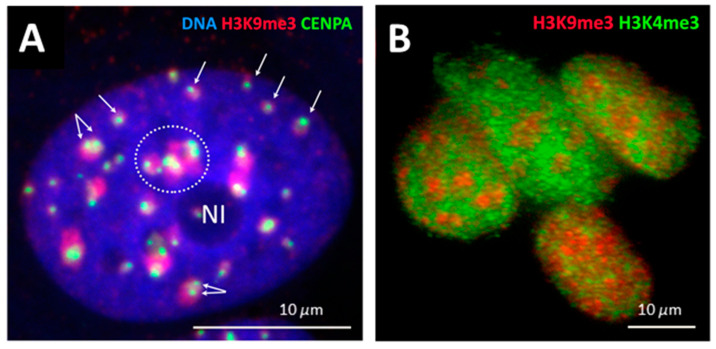
MCF-7 cells in chamber slides stained by immunofluorescence in 48 h starving control for: (**A**) PADs (H3K9me3), centromeres (CENPA), and DNA (NL—nucleolus) showing the unclustered and clustered centromeres in individual PADs (arrows and encircled); (**B**) PADs/H3K9me3 counterstained for active chromatin with H3K4me3 label. (**A**) Confocal and (**B**) epifluorescence microscopy. (Note: This figure was originally published under CC-BY license in [41]).

Our working hypothesis was that constitutive silencing pericentric chromatin domains (PADs) may change for the genome repatterning in differentiation commitment. We reproduced the model system of MCF-7 breast cancer cells treated with the ErbB3 ligand heregulin (HRG) with known dynamically traced transcriptome data [9]. PAD-repressive heterochromatin (H3K9me3), centromere-associated-protein-specific (CENPA), and active euchromatin (H3K4me3) antibodies were used for the microscopic image analysis on fluorescence and confocal microscopy. The typical staining patterns are presented in Figure 7.

**Figure 8:**
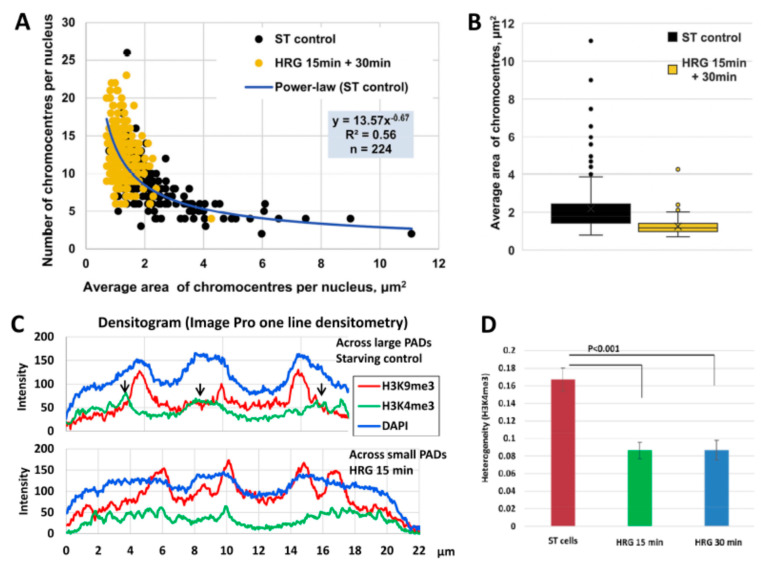
Influence of HRG treatment (15 and 30 min) on PAD distribution in ST (starving) control cells. (**A**) Relationship between the average number of PADs and their average area per cell; the distribution clearly shows the transition of both HRG 15- and 30-min cells covering only a small area in the ST population. The data of the three experiments are merged. ST control cells show a power-law distribution with high correlation (blue trendline); (**B**) box plots of the data presented in panel (**A**) showing high heterogeneity of the PADs in ST control cells and low heterogeneity and ~50% lower average area in HRG 15- and 30-min samples; (**C**) three-line mean densitometry examples: in the upper scan across large PADs in starving control and in the lower image across small PADs at HRG 15 min in the double-immunostained H3K9me3/H3K4me3 chromatin, counterstained with DAPI (recorded in corresponding optical channels), revealing in the upper scan the relatively dense H3K4me3 “collars” (arrows) at the borders and admixed in PADs included in larger DAPI-dense chromocenters, while in the lower scan the H3K4me3-positive material seems repulsed from PADS and distributed more evenly, decreasing the density of the DAPI chromocenters; (**D**) HRG treatment induces unfolding of the H3K4me3-positive chromatin. The results of the measurements of the heterogeneity parameter of the H3K4me3-stained, transcriptionally active chromatin are shown in the green channel using ImagePro Plus 4.5 program. After 15 min of HRG treatment, the active chromatin becomes 50% less heterogeneous (smoothened) and remains such at 30 min of HRG treatment. Averages of three independent experiments, with standard deviation. (Note: These figures and modified figure legends were originally published under CC-BY license in [41]).

In addition, an acridine orange DNA structural test was applied to test the conformation changing of the chromatin in the time course and showed the unfolding of the DNA occurring between 15 and 20 min after HRG action (for details, see [41]). The results of image analysis of PADs and active chromatin stained by immunofluorescence are shown in Figure 8.

The data presented in Figure 8 allow us to suggest that splitting the PADs under the threshold of silencing effect induces critical phase transition (by unfolding and repulsing) of the active genome compartment as a prerequisite of genome rewiring for starting cancer cell differentiation.

Moreover, the ability to split and fuse this constant number of PADs occurs mostly in a binary mode (1 + 1 = 2). The study revealed that the bursting of PADs to mostly split doublets coincided with the activation of the aspecific stress-response cassette (*FOS*, *FOSL*, *c-myc*), general DNA unfolding, and coincides with pushing the transcription avalanche of thousands of genes. This is likely needed for the change in cell fate allowing the commitment of differentiation, which occurs “in a jump” of about 5 min (15 to 20 min after HRG action), with erasing the positional information of the heterochromatin network.

In collaboration with Masa Tsuchiya, Kenichi Yoshikawa, Alessandro Giuliani and Giovanna Zimatore a chapter for a Springer book was written [42], finally bringing these observations together with long-standing considerations in the literature on genome computing [43]. In line with that reasoning, the optimal conditions for the support of computation needing transmission, storage, and modification of information are achieved by self-organization in the vicinity of a phase transition—at the edge of chaos. The organizational parameters of the constitutive heterochromatin, which was sometimes considered as “junk” DNA, change in space and time and thus can mediate—in addition to the linear genetic code—the control of the function of the genome by genomic computations, which is governed by the laws of thermodynamics of complex open systems.

### 2.4. The Circadian Clock and Cancer (Presented by N.M. Vainshelbaum)

**Figure 9:**
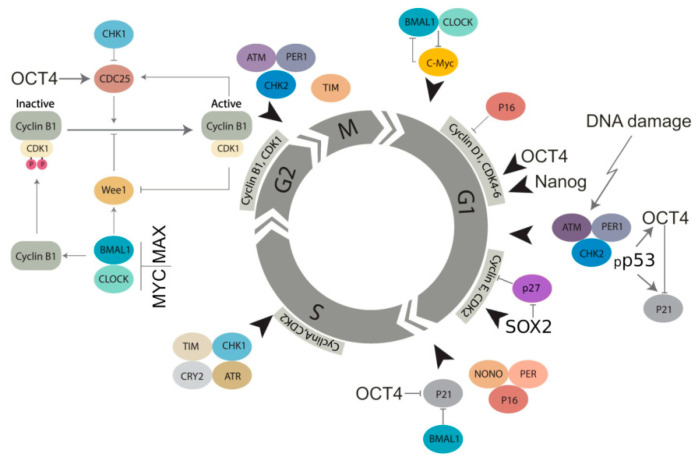
Regulation of the cell cycle and its checkpoints by specific cyclin-dependent kinases and the molecular components of the circadian clock. The role of stemness factors in adapting the normal cell cycle pace (Note: This figure was originally published under CC-BY license in [32]).

The core genes of the circadian clock (CC) directly interact with the components of cell cycle control machinery, regulating the checkpoints of the cell cycle and effectively pacing it [44]. The embryonic stem cell (ESC) transcription factors (*OCT4*, *NANOG*, *MYC*) enhance the activity of cyclin-dependent kinases (4,6,2) [45], thus competing against cyclin inhibitors, and also directly suppress the latter, speeding up the cell cycle to bypass the G1/S checkpoint (Figure 9). The CC is also involved in telomere negative-reciprocal regulation via the SIRT1-PER2 interaction [46,47,48]. It thus appears that the CC “counts” (shortens) the Hayflick limit of somatic cells (and by that, the organism’s lifespan as well).

Aggressive malignancy is associated with whole-genome duplication (WGD) and cyclical polyploidization, polyploid giant cancer cells (PGCCs) arising in response to the stress of treatment and later depolyploidizing and spawning resistant progeny that repopulate the tumor and lead to disease relapse [49]. Cancer polyploidy is linked to cell-fate change (a rewiring of the gene regulatory network to a condition of stemness with atavistic, embryonal and germinative properties). The similarity of a polyploid cancer cell to the state of an embryonic stem cell (tested by the expression of embryonic stem cells (germline) markers OCT4, NANOG, SOX2, and Myc) [50,51,52,53], as well as the fact that circadian deregulation is implicated in many diseases including cancer [54,55,56,57,58], warrants an investigation into the relationship between ploidy and the state of the circadian oscillator.

**Figure 10:**
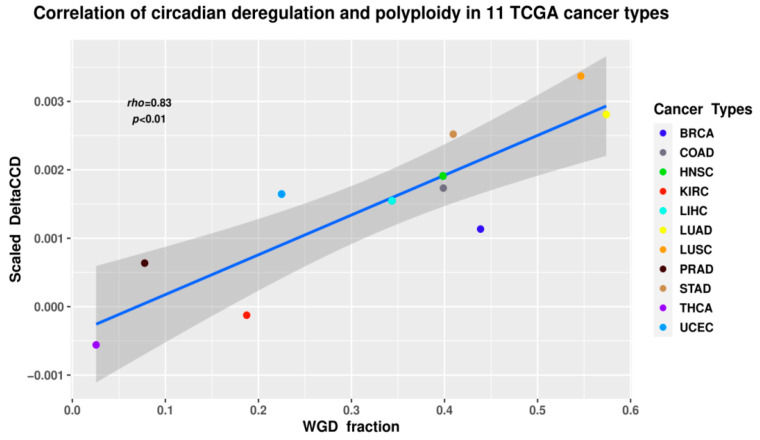
The DeltaCCD (clock correlation distance) coefficient (calculated using the method developed by [55]) describes the extent to which the core clock gene coexpression network diverges from its normal state. The tumor types with a higher prevalence of polyploid (WGD+) samples also demonstrate more profound CC deregulation (Spearman’s rho = 0.83, *p* < 0.01) and high disease aggressiveness: BRCA—breast carcinoma; COAD—colon adenocarcinoma; HNSC—head and neck squamous cell carcinoma; KIRC—kidney renal cell carcinoma; LIHC—liver hepatocellular carcinoma; LUAD—lung adenocarcinoma; LUSC—lung squamous cell carcinoma; PRAD—prostate adenocarcinoma; STAD—gastric adenocarcinoma; THCA—thyroid carcinoma; UCEC—uterine corpus endometrial carcinoma (Note: This figure was originally published under CC-BY license in [32]).

A study by Anatskaya and colleagues [59] identified downregulation of circadian clock-associated bivalent genes in normal endopolyploid tissues compared to diploid ones, while [32] extended those findings to 6054 cancer samples from the Cancer Genome Atlas database (Figure 10), identifying a strong positive association between polyploidy (inferred using the ABSOLUTE [60] algorithm from copy-number data) and the DeltaCCD coefficient, a metric of core circadian clock gene coexpression network deregulation calculated from RNA-seq data [55].

**Figure 11:**
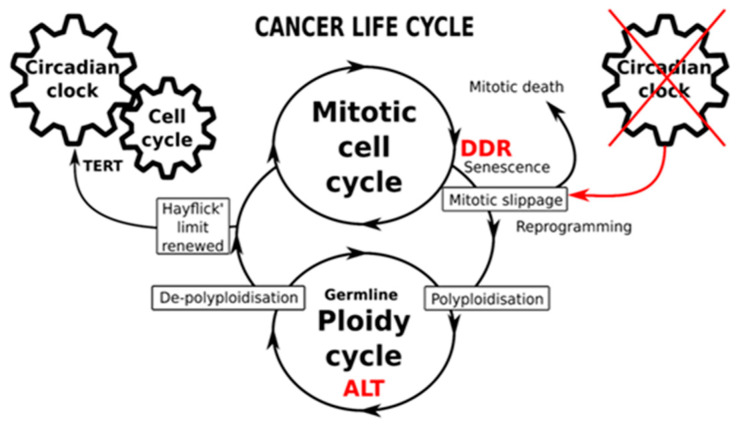
Mitotic cancer cells enter accelerated senescence caused by oncogenic, genotoxic or oxidative stress. DNA damage accompanying senescence activates the DNA damage response (DDR), whose checkpoint is adapted by “mitotic slippage” towards germline-related ploidy cycle (related to whole-genome duplication or polyploidization). For that time, the regulation of the cell cycle by the CC is lost. Recovery of telomeres and telomerase during the ploidy cycle (described by [53]) renews the Hayflick limit of the cell cycle pace by CC. Through this “CC death loop,” cancer cells can recover their immortality. (Note: This figure was originally published under CC-BY license in [32]).

These findings reinforce the similarities between advanced cancer and embryonic stem cells, and offer a possible explanation for a link between polyploidy, stemness and circadian deregulation. In order to polyploidize and persist after genotoxic therapy stress that induces DNA damage and senescence, cancer cells have to bypass the DNA damage response (DDR) checkpoints. As mentioned previously, circadian clock components regulate the cell cycle and the DDR. CC deregulation, which is thought to be induced by mitotic slippage, would thus serve both to induce and reinforce the PGCC state. Renewal of clock function, which necessitates the restoration of telomeres and telomerase function, would subsequently enable the return to mitotic division and tumor repopulation, with the Hayflick limit restored and cancer’s replicative immortality maintained. This hypothesis is schematically represented in Figure 11.

The finding concerning the relationship between polyploidy and circadian deregulation may have practical application in terms of both cancer prognosis and treatment. For example, the extent of circadian deregulation in the tumor could be measured in order to assess prognosis, or attempts to therapeutically “normalize” the circadian clock could be undertaken to impact the PGCC—a source of resistant tumor regrowth.

### 2.5. Spatial Relationship between Ribosomal and mRNA Transcription/Splicing Conveyor, Nuclear Lamin Rigidity, and Actin Cytoskeleton Tension (Presented by K. Salmina)

In the cell nucleus, transcription pulsing involves restructuring of the heterochromatin. The pulsing wave is transmitted from the nucleolus to nuclear speckles (spliceosomes), nuclear envelope, and translation sites in cytoplasm, presumably coordinating the conveyor of the ribosome biogenesis, messenger RNA transcription, splicing and protein translation causing responsive feedback, for adjusting transcription volume to the environmental demands. This is possible on the basis of the radial-concentric organization of the structural participants joining with the elastic cytoskeleton and sufficiently rigid nuclear lamin [36].

**Figure 12:**
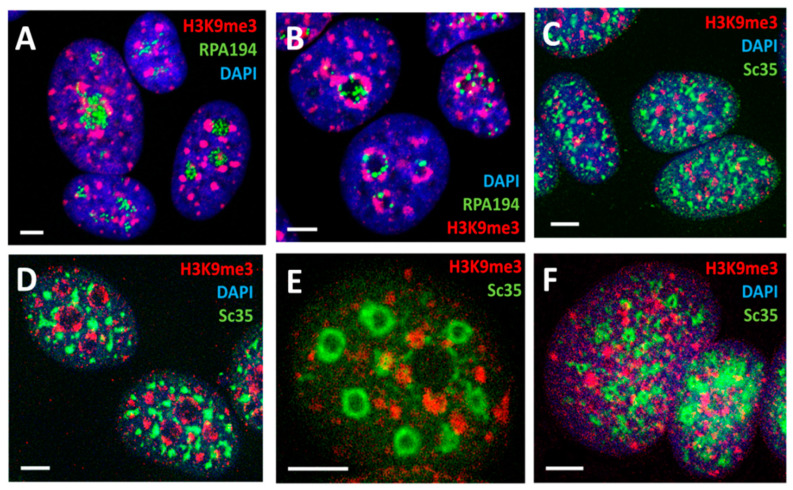
Partial inhibition of rRNA and mRNA synthesis by AcD and alpha-amanitin revealed the hidden radial-concentric spatial relationship between centers of nucleolar synthesis, perinucleolar PADs, and speckles; this order was destroyed after full suppression of transcription: (**A**) Control: the granules of RPA1 fill the nucleoli, indicating active rRNA synthesis; (**B**) AcD 0.2 µM/mL, 1 h—RPA1 form rare fused granules, indicating suppressed rRNA synthesis; H3K9me3 PADs compact and encircle the nucleoli; (**C**) control: elongated speckles and PADs seem disorderly distributed in the cell nucleus; (**D**) AcD 0.2 µM/mL, 1 h—clumped speckles surround the compacted shells of perinucleolar PADs; (**E**) alpha-amanitin 2 µM/mL for 2 h suppressing Pol II—an example of swollen empty speckles radially circumventing the deteriorating perinucleolar ring of PADs; (**F**) AcD 2 µM/mL for 5 h suppressing both RNA syntheses with chaotically distributed disarranged PADs and empty speckles. Scale bars = 5 µm. (Note: This figure was originally published under CC-BY license in [36].).

Nuclear speckles postulated as dynamic hubs of gene expression regulation and integrating the whole process of transcription [61] seem indeed capable of executing it through transcription pulsing of the chromatin network united with the actomyosin network. The topological relationship between the nucleolar fibrillar centers, perinucleolar heterochromatin, nuclear speckles, and lamin-associated heterochromatin after RNA transcription inhibition was studied. MCF-7 breast cancer cells were treated in chamber slides by increasing concentration/time of actinomycin D (AcD) and evaluated by immunofluorescence confocal image analysis. In control cells with active rRNA and mRNA synthesis, the perinucleolar repressive heterochromatin labeled by H3K9Me3/cen forms extended structures bent around nucleoli, speckles are located more externally and also extended, lamin B1 ideally outlines the nuclear envelope (NE), while actin filaments form fibrils both circular around NE and perpendicular or at angles to it, attached to the cellular membrane. When the nucleolar synthesis is initially suppressed by low concentration and short treatment with AcD, the remnant Pol I cofactor RPA194 forms a few large granules at the nucleolar margin, H3K9Me3 heterochromatin condenses in round clumps between them, and speckles also condense as regular dense and further circular structures around the latter, altogether revealing a radial-concentric nuclear pattern. After full suppression of RNA synthesis, the radial-concentric nuclear order is lost [36,62]. The typical patterns are presented in Figure 12.

**Figure 13:**
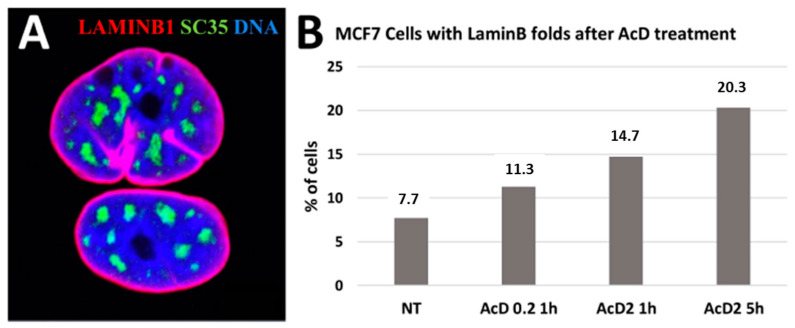
Softening of nuclear lamin testified as an increase of the proportion of cells with intranuclear lamin folds in the time course of suppression of RNA synthesis; (**A**) Nucleus without and with lamin B1 (evidenced by IF staining of lamin B1 (red) invaginations; (**B**) Counts of nuclei with lamin B1 invaginations after AcD treatment. (Note: This figure was originally published under CC-BY license in [62]).

The rigidity/elasticity of the nuclear envelope in the time course of RNA synthesis suppression was also studied. The lamin B1-positive nuclear contour becomes irregular and lamin B1 forms intranuclear channels often reaching the nucleoli, increasing in depth and frequency (~threefold) with deeper suppression of RNA synthesis (Figure 13).

**Figure 14:**
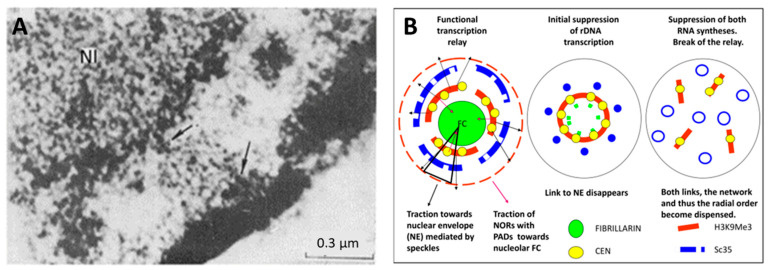
Speckles acting as radial spring pumps anchored between the nucleolar and perinuclear flexibly rigid heterochromatin shells and equipped by elastic actomyosin elements integrate the radial-concentric nuclear order for transcription pulsing. (**A**) Thin EM section of rat ascitic Zajdela hepatoma cells. The links of the speckle to the perinucleolar and perinuclear heterochromatin (arrows), Nl—nucleolus; (**B**) Schematic of the functional transcriptional relay in its relationship with speckles (SC35) and the concentric rings of the nucleolar and perinuclear heterochromatin shells (H3K9me3), deduced from experiments with suppression of RNA synthesis and the literature on the participation of the nuclear cytoskeleton in it. FC—fibrillar center, NORs—nucleolar organizers; CEN—centromeres. (Note: This figure was originally published under CC-BY license in [62]).

Electron microscopy of active cancer cells reveals a physical link of the nuclear speckles (earlier term “a cluster of interchromatin granules”) both with the perinucleolar and perinuclear (lamin-associated) heterochromatin (PADs and LADs, correspondingly) (Figure 14A). The literature data indicate the fibrils of actomyosin cytoskeleton become emerged by polymerization of f-actin from globular actin, simultaneously with activation of POLI, II, III and during splicing in statu nascendi, creating the functional, ordered cytoskeleton network of these processes [63,64,65]. Our studies showed that with partial suppression of rRNA synthesis, the perinuclear actin ring thickens (collapses onto the nuclear membrane, losing its rigidity), while its radial cytoplasmic fibrils become less tense (not shown; for details, see [62]). Full suppression of both syntheses by high dosage/prolonged AcD or a-amanitin results in full loss of the perinuclear actin ring and nuclear fixation in the cell center. The schematic of the proposed relationship between nuclear participants equipped with elastic actomyosin elements that integrate and elaborate in statu nascendi the radial-concentric nuclear order for transcription pulsing and collapse when RNA synthesis is suppressed is presented in Figure 14B.

We conclude that the links of the perinucleolar and lamin-associated heterochromatin with speckles intranuclear compartments are involved in topological coordination between the ribogenesis and mRNA synthesis and maturation, where the radial tension of the actin cytoskeleton exerted via concentric elasticity of the nuclear lamina forwards this conveyor through the nuclear pores towards translation sites. Our model simplistically presented in Figure 14B supposes that the nuclear speckles acting as radial spring pumps are anchored between the nucleolar and perinuclear flexibly rigid heterochromatin shells [36].

### 2.6. How Nanoscale Chromatin Architecture and Chromatin Topology within the Cell Nucleus Participates in Cancer Development—An Example of Pathogenesis of Three Different Leukemia Types (Presented by M. Falk)

#### 2.6.1. Chromatin and Cell Nucleus Architecture in Formation of Chromosomal Translocations—General Aspects

**Figure 15:**
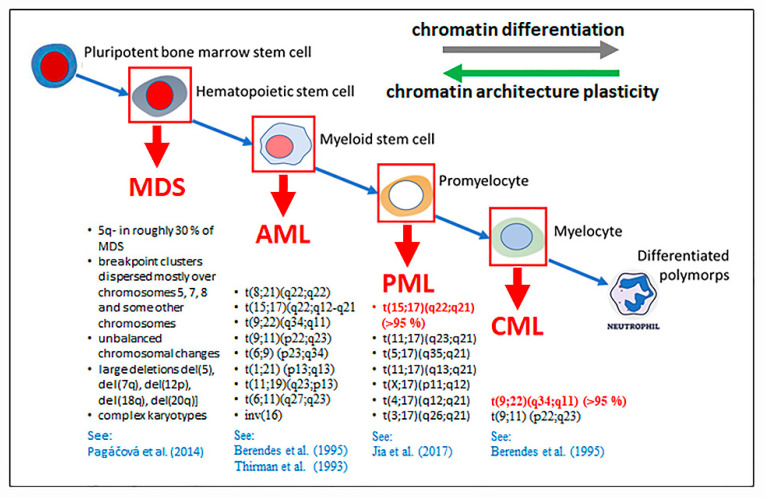
The relationship between the differentiation stage of particular leukemia cell type precursors, chromatin network plasticity of these precursors, and variability of genetic defects associated with a given type of leukemia [85,86,87,88]. Abbreviations: MDS: myelodysplastic syndromes, AML: acute myeloid leukemia, APL: acute promyelocytic leukemia, CML: chronic myeloid leukemia.

Leukemias are usually caused by one predominating chromosomal translocation, by a set of chromosomal translocations, or complex genotypes (Figure 15). Chromatin architecture, in many aspects specific to cell types, can be suspected to significantly contribute both to the more frequent occurrence of recurrent leukemogenic translocations and the variability of leukemias with respect to the spectrum of potential initiation defects of the genome. Important findings on the mechanism of formation of chromosomal translocations and the role of chromatin architecture in this process came from experiments where DSBs were generated at specific genetic loci in living cells by using DNA cutting enzymes [66] and radiobiological experiments, focused on DNA damage after exposure of cells to ionizing radiation (e.g., [67,68,69,70], reviewed in [71,72,73,74]). The results of these experiments are directly relevant for leukemias, as leukemias often appear secondarily as a side effect of radiotherapy of primary cancers or accidental irradiation. We learned that chromatin in the cell nucleus is organized according to certain rules at many hierarchical levels, although the resulting arrangement of chromatin is not strictly deterministic but statistical in nature [5,29,75,76,77,78]. The result of chromatin organization is thus a dynamic network of various chromatin domains with given structures and functions (reviewed in [5,79]). Internal organization of these domains and their arrangement in the cell nucleus is thus specific for cell cycle phases, cell types and level of differentiation and cell donor [80,81,82,83]. The positions of genetic loci in cell nucleus and, in turn, their proximity (e.g., within transcription factories) thus reflect all these holistic factors. Therefore, if some loci show a higher sensitivity to DSB induction (e.g., loci containing recognized fragile sites), and at the same time they are located close to each other in the nucleus, a DSB repair error can lead to the formation of translocations between these loci with a higher frequency than would correspond to chance [71,84].

If cells were irradiated with densely ionizing radiation or neutrons, concentrated energy deposition localized along in a small nuclear volume along the particle track [89] generated translocations mainly between chromosomes, whose interphase territories are often located in each other’s neighborhood [67,68,69,70]. Based on these findings, the so-called position or contact-first hypothesis has been formulated, which assumes that chromosomal translocations arise only between loci that had been sufficiently close to each other even prior to DNA breakage. The probability of a translocation occurring between two genomic loci is thus proportional to their spatial (3D) distance, given by a preset nuclear chromatin landscape. As discussed later, this preset genomic landscape is of statistical and dynamic character [29,75,76,77,78] and can be influenced by various factors, such as cell differentiation, disease, exposure to radiation or chemicals (stress), or age [90,91,92,93,94,95,96,97,98]. While the proportion of cells with spatially proximal *BCR-ABL* and *PML-RARa* loci was higher in hematopoietic precursors than in B-lymphoid cells, the cell type did not influence the proximity of *ABL* or *PML* genes and the beta-globin genes [70]. Chromatin re-organizations observed during differentiation may thus preferentially affect nuclear positions of genetic loci involved in recurrent leukemogenic chromosomal translocations and/or change the global chromatin landscape in a way supporting spatial interaction between these loci. Interestingly, *BCR-ABL* or *PML-RARa* transcripts were occasionally detected even in normal blood cells from healthy donors [99,100], suggesting that the evolution-optimized organization of the chromatin network has not avoided compromise situations where a certain organization of some components of this network is necessary for the physiological functioning of the genome and at the same time represents an increased risk in terms of the easy occurrence of chromatin network changes potentially leading to cell transformation. Moreover, *BCR-ABL* transcripts were more often observed in healthy males than females and the probability of their occurrence increased six times for adults when compared to children [99]. An important role of preset physiological chromatin architecture in formation of chromosomal translocations has been also supported by radiobiological studies showing that the overall mobility of chromatin at sites of DNA double-strand breaks (DSBs) is comparable to undamaged chromatin (e.g., [84,101], reviewed in [71,73,74]), provided chromatin is not highly fragmented by the application of densely ionizing irradiation [102]. Hence, according to the position-first hypothesis and many radiobiological studies, frequent oncogenic translocations arise as a result of the interplay of two factors: (1) the fragility of the respective loci (characteristic for a leukemia type) dictated by chromatin structure motifs at lower levels of organization, and (2) the nonrandom arrangement of chromatin in normal cells at higher levels of organization, given by spatial arrangement of structurally and functionally different subchromosomal domains and chromosomal territories [76,77,103,104,105]. Indeed, numerous DNA breakpoint clusters characteristic for different leukemias have been associated with previously known fragile sites [106]. In addition, genomic loci affected in leukemias were located in mutual proximity in a substantial fraction of cells isolated even from healthy donors [93]. For instance, the *BCR* (chromosome 22) and *ABL* genes (chromosome 9), involved in the formation of the Philadelphia chromosome—the molecular hallmark of chronic myeloid leukemia (CML)—were separated by only ∼0.3 or even 0.2 μm in 2% to 8% of healthy lymphocytes [93,107]. Similarly short distances were measured also between the *c-MYC* (chromosome 8) and *IgH* (chromosome 14) genes, in ~8% of human lymphocytes and fibroblasts [93]. This translocation can be found in ~98% of Burkitt’s lymphoma cases. Moreover, significant variations in *BCR-ABL* distances were revealed in bone-marrow cells of healthy individuals (and CML patients), suggesting that donor (person)-specific chromatin architecture may predetermine some individuals for the CML development [108].

Recently, the spatial proximity of frequently translocated partners has been studied with Hi-C techniques that overperform microscopy FISH (fluorescence in situ hybridization) visualization of potentially translocated loci in terms of resolution and throughput [109]. The results proved at the pan-genomic scale that gene partners involved in recurrent leukemogenic translocations often provide Hi-C contacts in normal, i.e., undamaged cells with intact chromatin [110]. In one of these Hi-C studies, Engreitz et al. [111] analyzed mutual spatial relationships between genetic loci involved in 1533 translocations reported in leukemia, solid cancers and germline genomes. The results proved that hundreds of translocation-prone partner loci, including loci involved in recurrent translocations in leukemia such as *BCR-ABL* and *MYC-IGH*, are spatially proximal in nuclei of normal human cells. Importantly, translocation partners reported in human hematologic malignancies showed more frequent Hi-C contacts in lymphoid cells than loci translocated in sarcomas and epithelial tumors, pointing to relevance of tissue- or even cell type-specific chromatin architecture in the development of chromosomal translocations. Nevertheless, as the translocation probabilities correlated with the frequency of Hi-C contacts in different tissues, it can be considered that both tissue-specific and universal features of chromatin structure participate in the mechanism of chromosomal translocation formation [111].

Indeed, the mechanism of chromosomal translocation formation is probably more complex, as is the role of higher-order chromatin architecture in this process. Complex chromosomal translocations containing three or more genetic loci are a characteristic type of chromosomal aberration in cells exposed to densely ionizing radiation (with high linear energy transfer, LET). In these cells, chromatin is pulverized along the trajectory of an energetic particle due to highly localized energy deposition [89,112]. Complex translocations can be thus formed even under the constraints given by the position-first hypothesis. However, explaining the occurrence of complex chromosomal translocations in cells irradiated with photon radiation of lower-density energy is difficult to explain by the position-first hypothesis. Photonic radiation deposits its energy homogeneously and the resulting DSBs are thus dispersed throughout the nucleus, i.e., mostly distant from one another. Thus, chromatin movement seems to be necessary for the formation of complex chromosomal translocations under given circumstances, which led to the postulation of the so-called breakage (movement)-first hypothesis. Supporting this hypothesis, high-throughput methods have proved in living cells that translocations can occasionally occur even between very distant (3D) genetic loci [66]. Whether this rare scenario can be limited to still-unknown cell states (e.g., aspecific stress) enhancing chromatin network plasticity [113] is unknown.

How these seemingly contradictory data can be unified into a more complex view on the mechanism of chromosomal translocation formation has been proposed in our previous works [71,74,84,114]. We showed that the overall chromatin mobility at DSB sites, quantified as the mean square displacement between all pairs of DSB loci, does not differ from undamaged chromatin in cells exposed to γ-rays. On the other hand, we identified a fraction of DSBs with significantly higher mobility, which were mostly located within condensed (hetero)chromatin domains. Changes of epigenetic markers observed at sites of highly mobile DSBs suggested that their movement can be related to decondensation of dense heterochromatin domains, which must occur as part of DSB repair [84]. The border areas of heterochromatin domains may thus represent nuclear sites with increased chromatin fragility [115]. An important step toward better understanding of the mechanism of chromosomal translocation formation was the finding that not only the distance between genetic loci but also the chromatin architecture (texture) between these loci influences, in a dominant manner, the probability of translocation. Enhanced plasticity of chromatin network, as it occurs, for instance, at the initiation stage of the cancer development and then again decreases in more advanced cancer cells or during the cell differentiation [113], could be another important factor promoting otherwise improbable mutual interactions between spatially distant genetic loci. Moreover, chromatin architecture also affects the sensitivity of structurally and functionally distinct chromatin domains, e.g., euchromatin and heterochromatin, to specific DNA damaging agents [91,116,117]. In brief, HP1 and potentially other structural proteins that specifically bound heterochromatin in high amounts shield DNA from free radicals generated by water radiolysis. Heterochromatin is thus better protected against the indirect damaging effect of ionizing radiation that is highly predominant for photonic radiation types [91,116,117].

Together, the results outlined above explain why some specific oncogenic translocations can be formed more often than corresponds to chance and significantly expand the role for chromatin architecture and cell nucleus organization in the mechanism of formation of chromosomal translocations.

#### 2.6.2. Roles of Chromatin Architecture and Its Plasticity in of the Development of Recurrent Chromosomal Aberrations Characteristic for Different Types of Leukemia

The involvement of chromatin architecture in the process of formation of chromosomal translocations (or other aberrations) has been predicted for decades; however, many questions even of a fundamental character remain open. An interesting question regarding the development of leukemias is why some types of leukemia are initiated by only one or several types of translocations, while other leukemias or preleukemic states are associated with multiple types of chromosomal rearrangements. As detailed below, chronic myeloid leukemia mostly (>95%) arise from one characteristic translocation, myelodysplastic syndromes (MDS) can be associated with very complex chromosomal rearrangements, although these rearrangements are still in some way characteristic of the disease in question.

Acute myeloid leukemia (AML) and chronic myeloid leukemia (CML) represent roughly 23% and 13% of all leukemia cases, respectively, and are illustrative examples demonstrating how chromatin architecture can participate both in leukemia initiation and expression of some leukemia-associated symptoms (discussed later). CML is caused by the clonal expansion of relatively “differentiated” myelocytes (Figure 15). At the molecular level, CML is determined very homogeneously by the reciprocal translocation t(9;22)(q34;q11), leading to the expression of the BCR/ABL1 oncogenic fusion protein that is expressed in about 90% to 95% of CML cases [118]. The translocation t(9;22)(q34;q11), known as the Philadelphia chromosome [119], and its product, BCR/ABL1 oncoprotein, originate from the erroneous end-joining between DSBs occurring within the *BCR* (breakpoint cluster region) and *ABL1* (Abelson murine leukemia viral oncogene homolog 1) genes, located on chromosomes 22 and 9, respectively. Though CML can be initiated also by some other translocation types, the DNA breakpoints most often appear within the two genomic regions—the band q34 of chromosome 9 and q11 of chromosome Moreover, DNA breakpoints are clustered also in the frame of the affected genes. Specifically, one breakpoint cluster exists between the exons 1 and 2 of the *ABL1* gene and three breakpoint clusters m-*BCR*, M-*BCR* and μ-*BCR* occur in exons 1, 13–15 and 19 of the *BCR* gene, respectively [119,120].

DNA breaks associated with translocations observed in acute myeloid leukemia (AML) are also clustered in sharp breakpoint clusters, similar to CML. For instance, the chromosomal breakpoints leading to the translocation t(8;21)(q22;q22) are located within the intron 5 of *RUNX1* gene and the intron 1 of *RUNX1T1* gene in most affected individuals [121]. Specific local chromatin architecture at lower organization levels thus seems to sensitize loci affected in AML to induction of DSBs. Supporting this hypothesis, eight of the eleven breakpoints detected in CML patients and 10 of the 14 translocation breakpoints detected in AML in the study of Gumus [106] were previously reported as the common fragile sites (CFS). The importance of chromatin architecture in the process of DNA damage is well illustrated also by the fact that the *BCR-ABL* translocation is not restricted to CML and AML, but has also been reported in a significant proportion of patients suffering from acute lymphoblastic leukemia (ALL) and, with lower incidence, also other hematopoietic cancers [122].

Additional evidence suggests that chromatin architecture at higher levels supports the formation of (recurrent leukemogenic) translocations in both CML and AML. Like CML, AML can also be caused by the t(9;22)(q34;q11) translocation [123]. However, in contrast to CML (and APL discussed below) genetic defects leading to AML are more variable, resembling more the situation with MDS (see Figure 15 and below). Unlike in MDS, though, the breakpoint loci are still sharply clustered in several short genomic regions, as it has been reported for CML (and APL). It can be assumed that both the position of chromosomal territories in the cell nucleus and the arrangement of chromatin within chromosomal territories determines the average distances between frequently mutually translocated loci [107]. It has even been found that when multiple types of oncogenic translocations can be involved in the initiation of a particular type of leukemia, chromosomal territories homing genetic loci potentially involved in translocations are more frequently located adjacent to each other in the cell nucleus than territories of nonparticipating chromosomes (reviewed in [107,124]. Interestingly, the length of the territory border between two interphase chromosomes has been shown to be a more important predictive parameter for the probability of the occurrence of a chromosomal translocation between given territories, than the extent of their chromatin intermingling [124].

However, some results disagree with the position-first hypothesis. For example, the mixed-lineage leukemia gene, *MLL*, forms translocations with up to 40 partners. A study of the mutual nuclear distances between the *MLL* gene, its frequent translocation partner *AF4* and the rarer partner *ENL* showed that the *MLL* and *ENL* genes are statistically closer to each other than the *MLL* and *AF4* genes. These surprising findings, however, do not necessarily challenge the validity of what we have written above. As has been suggested, local chromatin architecture at DSB site alters the mobility of the affected genetic locus. In addition, the architecture of the chromatin environment between the potentially translocated loci influences the probability of their mutual translocation. Together, these factors can favor either the aspects of the position- or breakage-first hypothesis (see [71] for a more detailed explanation).

In addition, it should be kept in mind that intracellular and environmental factors can modify the chromatin network arrangement and thus the formation of leukemogenic translocations. For instance, while leukemias occur spontaneously, their frequency, and thus the frequency of associated chromosomal translocations, dramatically increases after radiotherapy or chemotherapy of other primary cancers. Interestingly, we have demonstrated that irradiation forces the *BCR* and *ABL1* genes to move towards the center of the cell nucleus and become closer to each other [68,93,98,125]. Cell differentiation, senescence, aging, oxidative stress, initiated transformation and other phenomena have been shown to modify the architecture of the chromatin network and, in turn, can reasonably be expected to influence the formation of chromosomal translocations between specific genes.

To summarize, chromatin architecture at lower levels of organization seems to determine the susceptibility of specific chromosomal loci to DNA damage, while the organization of chromatin network in the cell nucleus is probably responsible for the more frequent interactions of genetic loci involved in recurrent leukemogenic translocations. In both these aspects, the roles of chromatin architecture seem to be similar in CML and AML. So why is CML associated with only one completely dominant translocation, while AML can be caused by a variety of different translocations [85]. The answer could be different plasticity of the chromatin network in CML and APL cells.

Cancer or precancerous cells in the initiation stage of transformation have been shown to dramatically increase the plasticity (i.e., the vulnerability to changes and the allowed scale of these changes) of their internal environment, including the plasticity of chromatin network [113] (genetic defects in precancerous cells have been recognized also in our previous work [97]). The plasticity then again decreases in advanced (metastatic) cancer cells [113]. We can hypothesize that the extent of changes in the chromatin network plasticity may depend on the stage of cell differentiation, where undifferentiated cells offer more room for plasticity than differentiated cells. Figure 15 demonstrates that the less differentiated cells a given type of leukemia originates from, the more types of chromosomal translocations can occur and lead to the development of the disease. Indeed, while CML arises from relatively differentiated myelocytes and is dominantly (>95%) caused by a single type of translocation, AML arises from much less differentiated myeloid stem cells and is associated with a variety of different translocations.

We can support the proposed hypothesis even more if we include other types of leukemia and preleukemic states in the discussion. Acute promyelocytic leukemia (APL) is in the vast majority (>95%) of cases initiated by the reciprocal translocation t(15;17)(q22;q21) between genes *PML* and *RARa* [86]. In rare cases, other translocation types have also been reported. However, most of these infrequent translocations share the common 17q21 DNA breakpoint, which is combined with one of the breakpoints occurring at several different locations in the genome [86] (Figure 15). The plasticity of the chromatin network and freedom in selecting the translocation partners in APL thus seem to be slightly higher than in CML, but lower than in AML. Accordingly, APL arises from promyelocytes, i.e., the blood cell differentiation stage upstream myoblasts (CML) but downstream myeloid stem cells (Figure 15).

Myelodysplastic syndromes (MDS) are a clinically and genetically heterogeneous preleukemic status subsequently progressing to AML [126]. In contrast to leukemia types outlined above, the molecular defects leading to MDS are highly variable all in the type of chromosomal rearrangements, genomic localization of DNA breakpoint “clusters” and extent of genomic architecture disturbance [87], though chromosomal rearrangements are not random, even in the case of MDS [87]. MDS is often initiated by a deletion occurring at the long arm of chromosome 5 (del(5q)), with other chromosomal defects gradually forming later and eventually ending up with highly complex genomes. MDS is also known for the occurrence of chromothripsis [127], a unique phenomenon in cancer development where one or more chromosomes are pulverized and randomly rejoined. Not surprisingly, in the context of these observations is the fact that MDS originates from the least differentiated cells within the mentioned leukemias.

Taking together, it can be predicted that the influence of chromatin architecture on the interactions between translocation partners has two components: (a) the “deterministic” component, resulting from the preset chromatin network organization in healthy cells and nonrandom reorganization of this network during physiological processes and in response to various impulses, and (b) the “stochastic” component, which can be described as random changes in chromatin network allowed by increased plasticity of this network during the early stages of carcinogenesis (or aspecific stresses?). The ratio of influence of these components may be related to the degree of differentiation of leukemic precursors. The contribution of the deterministic component is thus essential in CML and PML, while in AML and especially MDS, the stochastic component (of the genome stability) already contributes significantly. Verification of hypotheses proposed in the current manuscript represents an important future task.

**Figure 16:**
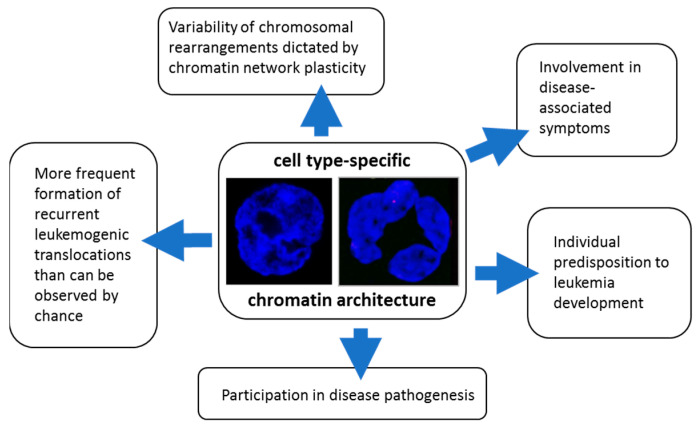
Multifaceted roles of the architecture of chromatin network in leukemia development and pathogenesis.

Finally, it should be emphasized that roles of chromatin architecture in the pathogenesis of leukemias are not restricted to the process of DNA damage and formation of chromosomal translocations (Figure 16).

Chromatin network organization and its alterations also directly participate in the pathogenesis of some of these diseases (e.g., APL, preliminary data available) or in the development of some associated symptoms, for example, immunodeficiency in AML/CML [96,128].

#### 2.6.3. New Insights into the Genesis of (Leukemogenic) Translocations at the Nanoresolution Level

Current nanoscale experiments can elucidate the relationships between DNA sequence motifs, the local structure of chromatin, and its susceptibility to spontaneous or induced damage. Superresolution microscopy (“nanoscopy”) can also further refine the spatial relationships between frequently translocated loci and the extent of their mobility in response to various stimuli or stressors. However, nanoscopy (e.g., single-molecule localization microscopy, SMLM) also opens completely new horizons of knowledge, for example, in the field of DNA repair [129,130,131,132,133]. Different mechanisms of DNA repair show different requirements for chromatin reorganization at the sites of its damage, have different reliability and therefore also different translocation potential [74]. A detailed understanding of these mechanisms at the microscale and nanoscale and the reasons for their activation at the sites of individual DNA double-strand breaks (DSBs) [74,134] are therefore critical for elucidating the process of chromosomal translocation formation in general and also with respect to specific translocation partners.

### 2.7. Schrödinger’s Chromosomal Aperiodic Crystal: A Model for Radiation Response of the Cell Nucleus (Presented by M. Hausmann)

In his presentation, Michael Hausmann (M.H.) went back to the early assumption of Erwin Schrödinger [135] that chromatin acts like an “aperiodic solid state within a limited volume,” and showed that the consequences of chromatin self-organization [136,137] in the limited volume of the cell nucleus are functionally determined networks [7] (e.g., heterochromatin network [36], network of ALU regions [138,139], network of L1 regions [139], etc.) and local topologies. Within these networks, chromatin architecture is rearranged during cell fate [41] and under environmental stress (e.g., radiation response) and interacts with epigenetic activities and feedback loops [140].

**Figure 17:**
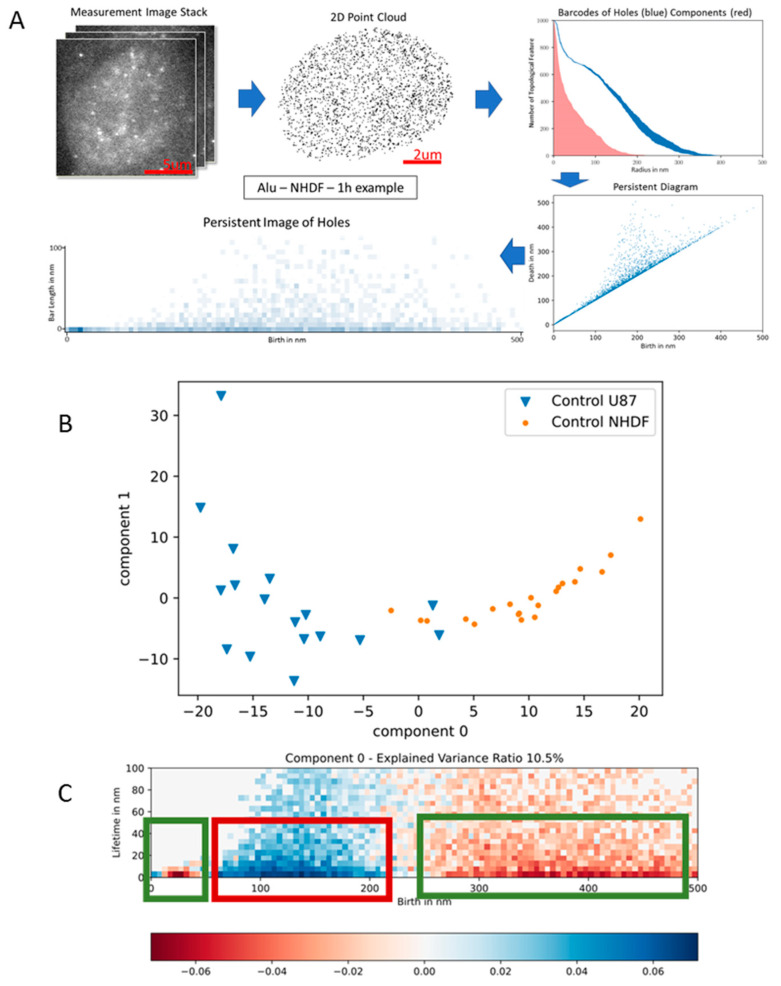
(**A**) An example of persistent homology workflow. After the measured image stack is converted into a 2D point cloud, persistent homology is applied. The results are represented as barcodes, showing each component (red) and hole (blue) as one bar. The persistent diagram, where each hole is shown as a point with birth and death as coordinates, is an equivalent representation. To vectorize the persistence diagram, it is converted into a persistent image by laying a grid over it and counting the holes in each grid cell. (**B**) After principal component analysis, the ALU networks of two cell lines can be separated and (**C**) the sizes of the holes of the network meshes can be determined (green rectangles: U87 glioblastoma cell line; red rectangle: NHDF fibroblast cell line).

In order to obtain quantitative values for chromatin networks, M.H. introduced the application of superresolution single-molecule localization microscopy (SMLM) [141], a method to determine the coordinates of single fluorescence molecules of specific labels within a cell nucleus with a precision in the 10-nanometer range [142]. These nanoscale coordinates are the basis for mathematical calculations of geometry and topology, e.g., Ripley distance frequency statistics of pairwise molecular distances [143], persistence homology [144], persistence imaging principal components analysis, etc. (see legend of Figure 17A).

With these approaches, it was shown that two cell types/cell lines (NHDF fibroblasts and U87 glioblastomas) can be distinguished by the topological characteristics of their ALU networks (Figure 17B). The ALU regions form a network [139] that is complementary to other networks [138], e.g., heterochromatin or euchromatin, and is known to be involved in chromatin (re)organization after irradiation causing DNA damage and repair [145,146]. In the example shown in Figure 17C, the ALU network in NHDF cells primarily forms meshes of about 100–200 nm “holes”, while the meshes in U87 show holes of about 300–400 nm diameter associated by dense regions of 20 nm holes.

After the exposure of cell nuclei to ionizing radiation (photon or particle radiation) inducing DNA damage, DNA double-strand breaks reorganize the chromatin architecture and seem to drive the appropriate repair process at a given damage site. Broken ends in heterochromatin cause heterochromatin relaxation [147] and are transferred to the border between euchromatin and heterochromatin by entropic forces [148]. The number of ALU network signals decreases with dose in a linear quadratic way [138,149], suggesting a molecular competition between remodeling protein binding and ALU oligonucleotide labeling probe binding.

**Figure 18:**
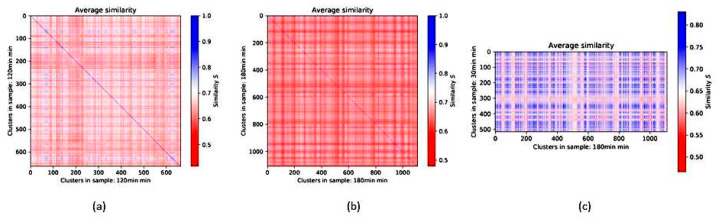
Averaged similarity heatmaps of γH2AX clusters of a fibroblast cell line. The values show a similarity measure based on the Jaccard index used to evaluate persistence homology data. The values vary between 0 (no topological similarity) and 1 (topological identity). Pairwise comparison of clusters (**a**) 120 min, (**b**) 180 min after irradiation. (**c**) Comparison of 30 min with 180 min clusters. Note: the differences in the color bars of the heatmaps comparing the same time point and comparing different time points. (Parts of this figure and a modified figure legend was originally published under CC-BY license in [133].).

Around a DNA double-strand break, phosphorylation of the histone H2AX to γH2AX takes place, which seems to have an impact on the chromatin arrangement of the broken ends [150]. As such, it was shown that γH2AX clusters are on average equally sized [151]. The topological similarity of these γH2AX clusters of heterochromatin origin is much higher as compared to clusters of non-heterochromatin origin [144]. Moreover, it was shown that the similarity of γH2AX clusters in early periods (about 30 min) after irradiation is in general higher than in later periods (for instance 24 h) because γH2AX clusters relax with successful repair. Nevertheless, if γH2AX clusters persist until later repair times, they maintain their topological similarity to their early formation. This can be shown by heatmaps using a similarity measure based on the Jaccard index (Figure 18) [133].

In the next step, M.H. showed that repair proteins like 53BP1 or MRE11 attach to the damage sites and form clusters related to the γH2AX clusters [132,133,152]. 53BP1 clusters cover or even overlap γH2AX; thereby 53BP1 cluster similarity seems to follow γH2AX cluster similarity. In contrast to 53BP1 which is strongly related to nonhomologous end-joining repair, MRE11 strongly related to homologous recombination repair forms small clusters integrated into the γH2AX clusters. On average, γH2AX forms topologically similar clusters in the early repair phase (up to 2 h), while MRE11 prefers to form similar clusters at later repair times afterwards.

In conclusion, the SMLM data presented in combination with geometric and topological analyses using mathematical approaches indicate cell type-characteristic network formation of different types of chromatin. The local characteristics of damaged sites and repair clusters are also network-like and indicate similarities depending on the origin region of the double-strand break. By these findings M.H. hypothesized that the characteristic size, shape and topology of damage and repair clusters contribute to the repair pathway decision a cell nucleus has to make at each damage site, especially between NHEJ (quick repair) and HRR (highly correct repair).

### 2.8. Significance of Non-Specific Physical Parameters on Genetic Activity through the Transition of the Higher-Order DNA Structure (Presented by K. Yoshikawa)

#### 2.8.1. Discrete Transition of Individual DNA

DNA molecules in viral capsids, bacterial nucleoids, and nuclei of eukaryotes occupy a volume 10-4–10-6 times less than they do when free in aqueous solution [153,154,155,156,157]. Whereas living organisms have elaborate apparatus for DNA packing such as nuclear proteins, a similar drastic decrease in volume to a compact/condensed state can be observed in vitro simply by adding various kinds of chemical agents, such as polyamine, multivalent metal cation, hydrophilic polymer, cationic surfactant, or alcohol [158]. Extensive studies on such drastic change in the conformation of DNA, known as “DNA condensation”, have been carried out. In the literature, “DNA condensation” had been frequently interpreted as a highly cooperative phenomenon, in other words, the transition had been regarded as steep but continuous [153,154,155]. It is also to be noted that most of the theoretical polymer physicists had kept the hypothesis that coil–globule transition should be always continuous until recently [159,160]. Nowadays, it has been clarified that, on many occasions, the seemingly continuous property of DNA condensation is attributable to the difficulty of the usual experimental methodologies to distinguish between the transition events occurring on individual DNA molecules and those of aggregation and precipitation of multiple DNA molecules [156,158,161]. It has been well established that the transition is an all-or-none type, or first-order phase-transition under the symmetry argument with order parameter in statistical physics, on the level of single giant DNA [156,158,161,162,163,164,165]. Theoretical analysis including the effect as a highly charged polyelectrolyte has reproduced the essential features of the coil–globule transition of DNA into a compact state in usual physiological solution condition containing ionic salts [166,167,168]. It is to be noted that a short DNA molecule of fewer than several hundred bp (base pairs) cannot fold itself because the persistence length of DNA is around 50 nm, which corresponds to 160 bp. Short DNAs aggregate without the generation of the folding transition. Interestingly, for the DNA molecules with the size larger than several dozen kbp, compact DNA exhibits a rich variety of morphologies depending on the solution conditions and kinetics [156,158,161].

#### 2.8.2. Inhibition/Promotion of Genetic Activity Accompanied by the Conformational Change of DNA

Here, the effect of conformational transition of DNA on the genetic activity caused by the addition of polyamines is described, by adapting the experiments with reaction solutions of cell-free gene expression. Polyamines are small multivalent cations that exist in all living organisms. It is well known that polyamines play important roles in many biological functions, such as cell proliferation, differentiation, apoptosis, and gene regulation [169,170,171]. They interact with negatively charged macromolecules such as DNA, RNA, and proteins, and thereby regulate the structure and function of these macromolecules. Polyamines induce the compaction/condensation of DNA, where higher-valence polyamines cause the folding transition at lower concentrations. From the measurements on the gene expression (transcription–translation, TX-TL, reaction) by the addition of polyamines, spermidine SPD(3+) and spermine SP(4+), it was found [172] that gene expression is increased by ca. fivefold with the addition of SPD(3+) at 0.3 mM, whereas it is completely inhibited above 2 mM. Similarly, with SP(4+), gene expression is maximized at 0.08 mM and completely inhibited above 0.6 mM. AFM (atomic force microscopy) observation revealed that a flower-like conformation is generated at polyamine concentrations associated with maximum expression. On the other hand, DNA molecules exhibit a folded compact conformation at polyamine concentrations associated with the complete inhibition of expression. It is expected that the enhancement of gene expression activity is due to the parallel ordering of DNA segments that is accompanied by a decrease in the negative charge of double-stranded DNA and that the inhibition is caused by the compaction of DNA into a tightly packed state with almost perfect charge neutralization [173,174,175].

#### 2.8.3. Longer DNA Exhibits Higher Genetic Activity

**Figure 19:**
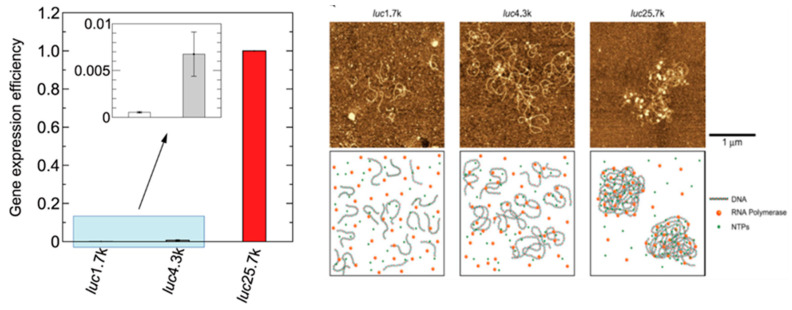
Left: Difference in the gene expression efficiency dependent on the size of template plasmid DNA. Right: AFM (atomic force microscopy) images of each linear reporter DNA in the reaction mixture for cell-free gene expression, together with the schematic representations on the bottom. (Note: This figure is a modified figure that was originally published under CC-BY license in [175]).

Adapting cell-free TX-TL systems, comparison of the efficiency of gene expression were performed for reporter DNAs encoding the firefly luciferase gene with different lengths [176]. Here, linear DNAs were adapted to make clear the effect of length-dependence in the absence of the effect of chain twisting. It was found that longer DNA molecules exhibit significantly greater potency in gene expression, for example, the expression level for DNA with 25.7 kbp is 1000 times that of DNA of 1.7 kbp (Figure 19).

AFM observation of the DNA conformation indicates that longer DNA takes shrunken conformation with a higher segment density in the reaction mixture for gene expression, in contrast to the stiff conformation of shorter DNA. Thus, it has become clear that such relatively long DNA molecules exhibit a shrunken conformation in the reaction buffer, which increases the opportunity for encountering RNA polymerase and NTPs due to an increase in their effective concentration around DNA. Although the discovery of the size effect of DNA on gene expression is still in a preliminary stage, it would be promising to extend this insight by shedding light on the actual mechanisms of gene regulation in living cells through extensive studies for longer DNA molecules with the sizes of Mbp–Gbp. It would also be interesting to examine the effect of DNA length on the activity of other enzymes, such as restriction enzymes, DNA repair enzymes, DNA polymerase, etc.

#### 2.8.4. Phase Segregation in Single DNA

As has been mentioned in I), individual duplex DNA chains above a length of several dozen kbp undergo a large discrete transition, or a first-order transition, from a spatially elongated state to a collapsed folded state (coil–globule transition) with the addition of various condensing agents. It is noted that the ensemble average of the effective size, radius of gyration or radius of hydrodynamic radius of DNA appears to be continuous since the relative population between coil and globule, changes gradually in the coexistence region according to Boltzmann’s law. In other words, an all-or-none change in conformation of individual chains is caused under the condition that the correlation length in the phase transition is greater than or equal to the chain length. Thus, when the chain length is above the correlation length, intrachain phase-segregated state, i.e., coexistence of a coil state and collapsed state in an individual molecular chain, is generated as a stable state. In fact, our studies using single chain observation confirmed the appearance of the intrachain phase-segregated state from a single DNA above the length of several dozen kbp [177,178,179]. As for the cause to generate phase segregation in a polyelectrolyte chain or shortening of the correlation length of the compact state, Coulombic instability may work under the condition of low salt concentration in solution. In other words, competition between long-range Coulombic repulsion and short-range attraction induces intrachain phase-segregated conformation. On the other hand, for the usual physiological aqueous solution both for in vitro and in vivo, the effect of coexisting counter ions should play the main role to cause the intrachain phase segregation, as has been discussed theoretically by incorporating the contribution of translational entropy of the counterions for free energy [179,180,181]. From the experimental side, it has been shown that chromatin reconstituted from 106 kbp plasmid and core histones undergoes a folding transition from an elongated state into a compact state accompanied by the generation of a phase-segregated state [182]. It is noted that the stability of such a phase-segregated state in reconstituted chromatin is sensitively dependent on the experimental parameters such as density, presence/absence of histone tail, salt concentration, etc. [183,184].

#### 2.8.5. Importance of Aspecific Parameters

According to the usual textbooks of biochemistry and physiology, it is regarded that living cells are controlling themselves by using complicated network of key-lock interactions. In other words, key–lock interaction has been regarded as robust. Based on the consideration of statistical thermodynamics, key–lock interaction is characterized with rather large stabilization of the bound state, i.e., equilibrium binding constant is large enough to stabilize the interaction. This picture may be reliable for a large ensemble of keys and locks. However, on a single-molecule level, such interaction should be under large thermal fluctuation. Thus, it may be obvious that robust self-control of living cells such as differentiation could not strictly control the cellular system in an on/off two-state manner owing to the fluctuation characterized by k_B_T. On the contrary, first-order phase transition as in the case of folding transition of genome-sized DNA is rather robust against the thermal fluctuation. Actually, recent studies reveal the rather significant effect on the structure and activity of DNA molecules above the size of several dozen kbp. For example, recently, it was found [185] that K^+^ enhanced gene expression in the presence of spermidine, SPD(3+), much more strongly than Na^+^, through in vitro experiments with a luciferase assay on cell extracts. Single-DNA observation by fluorescence microscopy showed that Na^+^ prevents the folding transition of DNA into a compact state more strongly than K^+^. ^1^H NMR measurement revealed that Na^+^ inhibits the binding of SPD to DNA more strongly than K^+^. Thus, SPD binds to DNA more favorably in K^+^-rich medium than in Na^+^-rich medium, which leads to favorable conditions for RNA polymerase to access DNA by decreasing the negative charge. The large difference in the effects of Na^+^ and K^+^ on in vitro gene expression is attributable to the different interference effects of these monovalent cations on the binding of polyamine to DNA, at least as the main cause. Further studies on the biological effects of the competition between monovalent cations and polyamine will shed light on the longstanding unsolved problem concerning the selectivity between Na^+^ and K^+^ in living systems.

As for the possible importance of the aspecific parameters, it has been shown that concentration changes of RNA [186,187], NTP [188], polyamines [189,190] and solvable macromolecules [191] cause on/off switching on transcription. In conclusion, it is highly expected that cooperation between key–lock interactions and aspecific interactions is constituting the basis of spatiotemporal self-regulation for gene regulation, in accordance with the field hypothesis of self-organization in life systems [23,192].

### 2.9. Insights from Alignment-Free Analyses of DNA Data Bases (Presented by G. Hildenbrand)

The potential of chromatin response to environmental stress and cell-fate change, e.g., chromatin reorganization under given conditions, must already be included in the DNA sequence by components and motifs preferring certain organizational structures. In order to elucidate such motifs and their positioning in the genome, DNA databases of sequenced species are investigated by statistical means.

Several types of regulation of gene activity are already known, as e.g., modifications by methylation of DNA in order to silence genes by methylation of CpG-dense promoters [193,194] or by repression of transposable elements [195,196], by modification of histones [197,198,199], the regulation via available transcription factors [200,201] or the regulation of RNA abundance by RNA interference [202,203,204,205].

All these factors influence directly or indirectly the 3D chromatin structure and are also depending on it. From a biophysical perspective, the 3D chromatin structure may also depend on a very basic level fundamental biophysical properties of DNA, such as, e.g., the base composition. There are already insights showing interesting functions of chromatin related to physical properties based on code [206,207,208]. Some hints have been found that demonstrate physical properties are encoded in short DNA words [209,210]. This may be reframed as “interesting functions of chromatin depend (also) on short DNA words”.

The general concept of finding something interesting, if it is not known what it really is searched for in DNA, may be as follows:

(1) Search: Find conserved genomic DNA patterns;
(2) Analysis: Look for association with known functional elements. Look for properties of identified DNA sequences;
(3) Interpretation: Make conclusions on function of found DNA patterns.

**Figure 20:**
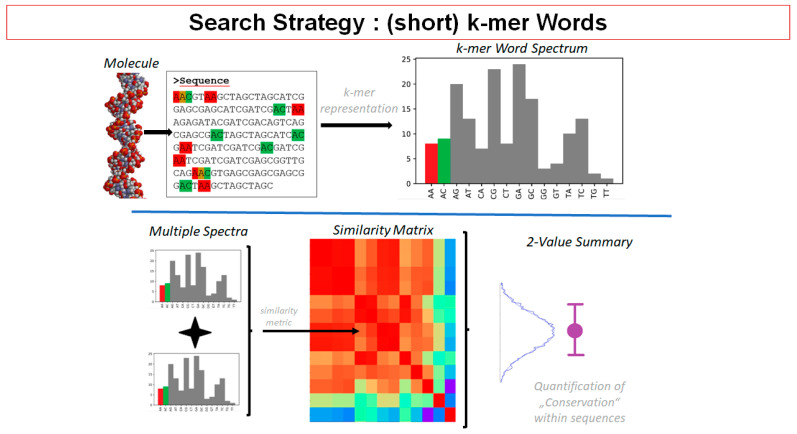
Showing the first steps of the search strategy and analyses for k-mer words. After sequencing, the k-mers are searched for, counted, spectra of abundances of abundances are in pairs correlated and may be given in a heatmap with color-coding for the correlation value. This may be further condensed by averaging all the correlation values and giving the distribution with mean and also deviation.

The first approach in this field may be done by searching for k-mers (all the possible substrings of length k) and analyzing their abundance and also distribution, as schematically shown in Figure 20.

Results found by this approach are given and also discussed in [211] for a eukaryotic approach, showing that k-mer abundance seems to be highly conserved especially in intronic and intergenic regions for large subsets of eukaryotic genomes and with hints for a strong impact of k-mers structured by repeat units of repeat length 1 bp or 2 bp. The strongest contributions may be seen from those with 1 bp repeat unit length, but AT or TA as base for 2 bp repeat units seem to be very strong in some kingdoms (as Embryophyta and Protista) and some as GT/AC appear as specific features of Animalia).

This encourages the search for a specific subset of k-mers, named SSTRs (supershort tandem repeats) with exact length in bp and no possible elongation as for k-mers in a sliding window approach.

The combination of position information of the SSTRs with their abundance as in principle shown in [212] by mapping of SSTRs reveals specific patterns in centromeric and other regions and are also depending on the length of the SSTRs and may show also correlations with specific sites for mutations [manuscript in preparation].

**Figure 21:**
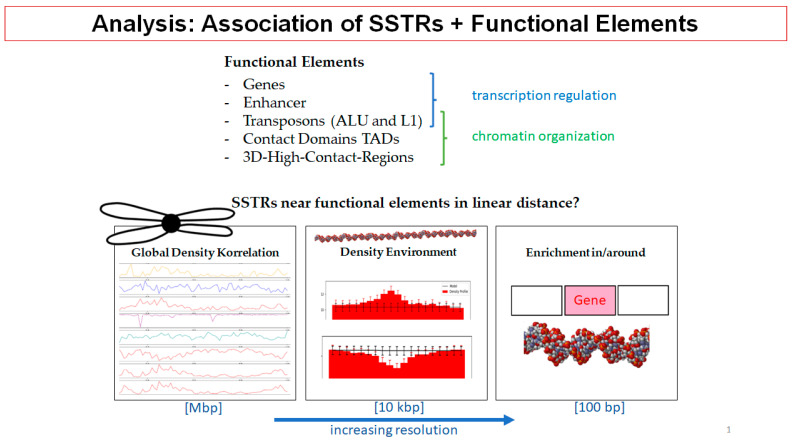
The correlation of SSTRs along a DNA sequence may be assessed with several functional elements, e.g., genes or transposons and on several levels of resolution along the chromosomal sequence. (Note: This figure is a modified figure that was originally published under CC-BY license in [209]).

The association of SSTRs with known functional elements may be done on several levels as shown in Figure 21. The results of these associations show specific properties depending on length and composition of the SSTRs and on the very type of genes and transposons (Henn et al., manuscript in preparation). Comparing these findings with known biophysical parameters, e.g., roll or slide, shows correlations with SSTRs and also depending on length and composition (A. Sievers et al., manuscript submitted). From these findings we may consider the following:

(1) Conserved patterns based on k-words and SSTRs are found in genomes;
(2) Strong correlations/anticorrelations with biophysical parameters are found;
(3) Organization of DNA may not only be based on long conserved sequences but also on base composition and length of SSTRs.

## 3. Summary and Conclusions

In this article, we have taken up considerations and investigations that were presented at the international symposium Spatial–Temporal Genome Regulation in Stress-Response and Cell-Fate Change in Riga, July 2022. Organization principles shown for biological systems are based on the concept of self-organized-complexity (SOC), a fundamental principle deriving from statistical mechanics that can be applied in a wide range of scientific investigations and models. This results in a strong impact of positional information of chromatin in time and space on genome regulation. Changes in cell activities and cell fates were found to be associated with phase transitions of chromatin organization that correspond with oscillations of protein expression. Under normal conditions, all these activities seem to follow a circadian clock, which fails in case of cancer development through polyploidy. Thereby, it has been shown that this is also causally related to changes in chromatin organization. A major organization principle forced by self-organization under boundary conditions of a limited volume is the formation of networks for chromatin of the same type (heterochromatin, Alu regions, etc.). Such networks reflect different specialization of cell types and react to environmental stress by characteristic changes. On a very coarse-grain level, the ubiquitous presence of scale-free structural units in many of the studied phenomena suggests a cooperative “control architecture” based on networks of interacting elements to produce emergent behavior at higher scales. This happens at both functional (gene expression) and structural (chromatin organization) level and encompasses the classical inventory of complex system dynamics made of multiple attractor states and phase transitions.

The DNA sequence counterparts of these rich dynamics are still obscure, but the presence of appropriate sequence motifs (some of retroviral origin and behaving as toggle-switch controllers) are likely to constitute crucial players of the game.

It is worth noting that the physics of biological regulation is still in its infancy: we can state some general organizing principles are active at different scales, not allowing for detailed quantitative predictions, but sufficient to detect “tipping points” of transition behavior [213,214]. This opens the perspective of the foundation of a sort of biological statistical mechanics that promises to be a turning point in the development of biology as a quantitative science.

The symposium showed the broad facets of current research on the cell nucleus as a holistic system, but also showed how many questions remain to be answered. With novel techniques and methodological approaches, many tools are on hand that will bring us towards better understanding what happens behind cell reactions in future investigations. That also means that deeper understanding of the Physics of Life should become an important part of the biomedical education curriculum.

## Data Availability

Not applicable.
